# Cardio-Oncology Risk Phenotypes in Cancer Patients Treated with Chemotherapy: A Data-Driven Analysis

**DOI:** 10.3389/fcvm.2026.1826258

**Published:** 2026-08-03

**Authors:** Xiaolin Gao, Kexin Chen, Haowen Liang, Andong Huang, Qiexinhao Li, Jianpeng Liang, Qiutong Pan, Ruilang Zhong, Jiayao Zhang, Ruirui Liang, Weipeng Zheng, Kenie Wang, Jun Peng, Tao Yang

**Affiliations:** 1 Department of Anesthesiology, The Second Clinical College of Guangzhou Medical University, Guangzhou, China; 2 Department of Clinical Medicine, The Nanshan Clinical College of Guangzhou Medical University, Guangzhou, China; 3 Department of Clinical Medicine, The Third Clinical College of Guangzhou Medical University, Guangzhou, China; 4 Department of Clinical Medicine, The Second Clinical Medical College, Southern Medical University, Guangzhou, China; 5 Department of Clinical Medicine, The Sixth Clinical College of Guangzhou Medical University, Guangzhou, China; 6 Department of Clinical Medicine, The First Clinical College of Guangzhou Medical University, Guangzhou, China; 7 Department of Orthopaedics, School of Medicine, The Second Affiliated Hospital of South China University of Technology (Guangzhou First People's Hospital), Guangzhou, China; 8 Shengli Clinical Medical College of Fujian Medical University, Department of Breast Surgery, Fujian Provincial Hospital, Fuzhou University Affiliated Provincial Hospital, Fuzhou, China; 9 Department of Cardiovascular Medicine, The Second Affiliated Hospital, School of Medicine, The Chinese University of Hong Kong, Shenzhen & Longgang District People's Hospital of Shenzhen, Guangdong, China

**Keywords:** cancer survivors, cardio-oncology, cardiovascular disease, chemotherapy, machine learning, risk phenotyping

## Abstract

Cardiovascular disease (CVD) is a major long-term complication in cancer survivors treated with chemotherapy, yet risk varies markedly across individuals. Using the UK Biobank, we studied chemotherapy-treated cancer patients without baseline CVD and identified data-driven cardio-oncology risk phenotypes with unsupervised clustering (UMAP embedding and HDBSCAN). We evaluated associations between phenotypes and incident CVD using Cox proportional hazards models and assessed 5-year risk discrimination with and without phenotype labels. Among 18,693 patients, 9 stable phenotypes were identified, characterized by distinct patterns of cancer treatment exposure, baseline cardiovascular medication use, cardiometabolic burden, renal function, and lifestyle factors. Compared with the reference phenotype, all phenotypes had higher risks of composite CVD, and the highest-risk phenotype demonstrated a hazard ratio of 4.06 (95% CI, 3.44–4.78). Phenotype-defining features were interpreted using SHAP values. In subtype analyses, heterogeneity was most apparent for ischemic heart disease, angina pectoris, and acute myocardial infarction. Adding phenotype labels modestly improved 10-year discrimination, with AUC values increasing from 0.648 to 0.692. These findings suggest that phenotype-based profiling may provide an exploratory framework for risk stratification in cardio-oncology and may help characterize risk heterogeneity across cancer survivors treated with chemotherapy.

## Introduction

Over recent decades, advances in early cancer screening and improvements in systemic therapies, including chemotherapy and targeted treatments, have substantially increased overall survival among cancer patients worldwide (1, 2). However, as survival has lengthened, an increasing number of cancer patients have developed treatment-related chronic complications (3). Multiple large-scale epidemiological studies have consistently shown that cancer survivors face a substantially higher risk of cardiovascular events and non-cancer mortality compared with the general population, with this risk exhibiting pronounced heterogeneity across different age groups and cancer types (4–8). Consequently, cardiovascular disease (CVD) has emerged as one of the leading non-cancer causes of impaired quality of life and adverse long-term prognosis among cancer survivors, particularly in non-metastatic cancers, where prolonged follow-up reveals that CVD-related mortality may exceed cancer-specific mortality for certain tumor types (9). Chemotherapy-related cardiovascular injury can occur through multiple mechanisms, including oxidative stress, mitochondrial dysfunction, inflammatory activation, and endothelial impairment (10–13), ultimately resulting in adverse outcomes such as heart failure, ischemic heart disease, arrhythmias, and stroke (14, 15). Notably, such injury often persists beyond the completion of cancer therapy, thereby conferring sustained long-term cardiovascular risk (14). In response to this growing clinical burden, the 2022 European Society of Cardiology (ESC) cardio-oncology guidelines explicitly recognize cancer patients as a population at lifelong increased cardiovascular risk and recommend structured, long-term cardiovascular surveillance. This consensus underscores the critical importance of strengthening risk identification and stratified management of treatment-related cardiovascular complications alongside improvements in cancer survival.

Previous studies have established that chemotherapy is associated with an increased risk of cardiovascular events (16, 17). However, most existing research has focused on specific drugs or individual cancer types (16), with limited efforts to systematically evaluate cardiovascular risk across the overall population of patients receiving chemotherapy. In addition, most current risk models are based on average-effect assumptions and therefore fail to capture the substantial heterogeneity of cardiovascular risk within chemotherapy-treated populations. Even among patients receiving similar chemotherapy regimens, cardiac functional responses vary markedly between individuals (18, 19). Moreover, patients undergoing chemotherapy differ substantially in metabolic status, renal function, and inflammatory status. Traditional risk models rely on a limited set of linear predictors and are often unable to capture the complex interactions between multidimensional physiological characteristics and treatment exposures. As a result, some high-risk subgroups may be systematically underestimated. Evidence from the global Prospective Urban Rural Epidemiology (PURE) study, which included participants from 26 countries, showed that approximately 41% of cardiovascular events occurred in individuals classified as low risk by conventional models. This finding suggests that current risk stratification approaches may underestimate the true burden of risk in certain vulnerable subpopulations (20). Consequently, accurate risk stratification and comprehensive assessment of chemotherapy-related cardiovascular toxicity remain unresolved challenges in cardio-oncology.

In recent years, unsupervised machine learning methods have been increasingly applied to disease phenotyping and precision risk prediction, offering new approaches to disentangle clinical heterogeneity in complex diseases. A growing body of evidence has demonstrated the utility of these methods in conditions such as heart failure, stroke, and diabetes (21–23). Studies across multiple disease domains have demonstrated that clustering approaches based on multidimensional features can identify patient subgroups with distinct biological mechanisms and prognostic trajectories. However, this strategy has not been systematically applied to explore heterogeneity in chemotherapy-related cardiovascular risk. Against this background, leveraging the large-scale prospective cohort of the UK Biobank, we developed an interpretable machine-learning-based phenotyping framework that integrates demographic characteristics, metabolic status, and chemotherapy exposure to identify latent cardio-oncology phenotypes among cancer patients after chemotherapy. This approach aims to enable early identification of high-risk subgroups and more precise cardiovascular risk assessment. By uncovering the underlying heterogeneity of post-chemotherapy cardiovascular risk, our study seeks to provide a novel conceptual basis and risk stratification strategy for the long-term cardiovascular management of cancer patients.

## Methods

### Study population

**Figure 1:**
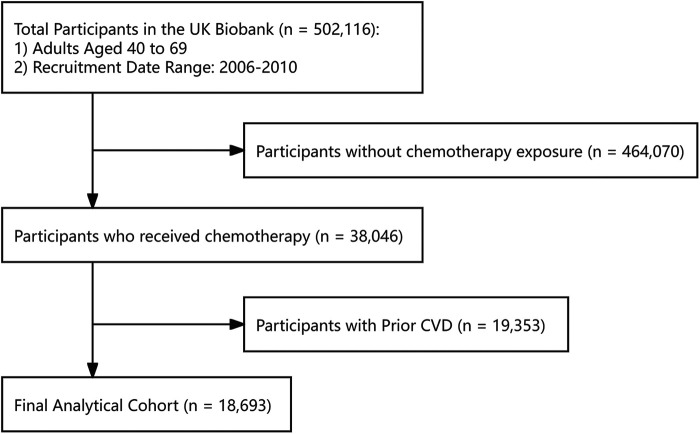
Participant flowchart for the UK Biobank cohort. CVD, cardiovascular disease.

In this study, we used data from the UK Biobank, a large prospective cohort study that recruited approximately 500,000 participants (40–69 years old) through the National Health Service (NHS) between 2006 and 2010. Follow-up was censored on October 31, 2022, for England; July 31, 2021, for Scotland; and February 28, 2018, for Wales (24). This research was conducted under UK Biobank application number 143798 and received ethical approval. All participants signed informed consent forms. After excluding individuals with CVD prior to chemotherapy diagnosis, 18,693 eligible participants were ultimately included. Missing baseline covariates were not used as exclusion criteria and were subsequently handled using multiple imputation. The study design and exclusion criteria are detailed in Figure 1.

### Ascertainment of cancer and cardiovascular disease

In this study, exposure was defined as a known history of chemotherapy. The primary outcome was incident CVD (ICD-10 codes I00-I99), Secondary outcomes included specific subtypes such as hypertension and ischemic heart disease (Supplementary Table S1). Follow-up began at the date of the first chemotherapy record and ended at the occurrence of a first CVD event, death, or the end of follow-up.

### Covariate collection

In the UK Biobank cohort, 45 variables were used for clustering. These variables covered basic clinical information, lifestyle factors, metabolic status, and inflammatory biomarkers. Additionally, cancer treatments (chemotherapy and radiotherapy) and medication use (antihypertensive, lipid-lowering, and glucose-lowering drugs) were also included. These variables are routinely collected during the clinical assessment of patients receiving chemotherapy or have been proven to be significant for CVD risk stratification and prognosis (25–27). All variables and their field IDs are listed in Supplementary Tables S1, S2. Age was defined as age at baseline recruitment (UK Biobank field ID 21022), and all baseline covariates were obtained from the initial assessment visit.

### Unsupervised clustering of baseline clinical and treatment-related features

An unsupervised clustering framework was applied to identify patient subgroups based on key clinical features. Similarity between individuals was quantified using Gower distance, followed by nonlinear dimensionality reduction using uniform manifold approximation and projection (UMAP), with 50 nearest neighbors and a minimum distance of 0.1. Clusters were identified using Hierarchical Density-Based Spatial Clustering of Applications with Noise (HDBSCAN) with a minimum cluster size of 500, and samples classified as noise were excluded. Sensitivity analysis was performed using bootstrap resampling with Jaccard similarity, and comparisons with K-means, hierarchical clustering, and partitioning around medoids (PAM) using the adjusted Rand index (ARI) and normalized mutual information (NMI).

### Statistical analysis

Missing baseline covariates were handled using multiple imputation by chained equations (MICE) under the missing-at-random assumption. Imputation was performed before feature selection, clustering, and survival analyses to reduce potential bias and loss of efficiency associated with complete-case analysis. Continuous variables are represented by the mean and standard deviation, while categorical variables are represented by frequency and percentage. We applied LASSO regression to 36 candidate baseline variables to obtain a stable set of features for subsequent clustering and prognostic analysis. We then applied outcome-informed LASSO-Cox regression to candidate baseline variables, incorporating follow-up time and incident CVD status, to select a stable set of features for subsequent unsupervised clustering and prognostic analysis. After clustering based on these features, we used SHAP (SHapley Additive exPlanation) to explain the main feature contributions of different subgroups and employed a Cox proportional hazards model to assess the risk association between each subgroup and total CVD and each subtype of event. To evaluate the incremental predictive value of cluster information, we constructed two predictive models. Model 1 included established CVD risk factors: age, sex, race, smoking status, alcohol consumption, body mass index (BMI), physical activity (MET-minutes/week), sleep duration, Townsend deprivation index, blood glucose, glycated hemoglobin (HbA1c), total cholesterol, high-density lipoprotein (HDL) cholesterol, triglycerides, C-reactive protein (CRP), and serum creatinine (28–30). Model 2 added cluster labels to Model 1 to assess the independent gain of subgroup information on risk prediction. Model performance was assessed using 5-fold cross-validation, including the area under the curve (AUC) at 5 years, time-dependent AUCs during 3–10 years of follow-up, the Brier score for calibration, and Harrell's C-index (concordance index) for overall discrimination. All statistical analyses were performed using R version 4.4.3 (Vienna, Austria). A two-sided *p*-value of less than 0.05 was considered statistically significant.

### Characterization of noise-classified participants

Because HDBSCAN identifies both core clusters and noise points, we additionally compared the baseline characteristics of noise-classified participants with those of the core clustered population to improve transparency regarding participants not assigned to any core cluster and to assess the potential for selection bias associated with noise exclusion.

### Sensitivity analyses

To assess the potential influence of sex on cluster formation, we performed additional sensitivity analyses using the same analytical pipeline as in the primary analysis. First, we repeated the clustering analysis after excluding sex from the clustering input variables, while retaining the other selected features and re-applying the UMAP-HDBSCAN procedure. Second, we conducted sex-stratified clustering analyses separately in female and male participants using the same clustering procedure within each subgroup. In both analyses, identified clusters were subsequently characterized according to their baseline clinical features, and Cox proportional hazards models were used to evaluate associations between cluster membership and cardiovascular outcomes.

## Results

### Cohort selection and baseline characteristics

During a median follow-up of 6.06 years (interquartile range, 1.56–10.34), outcomes were systematically ascertained. The baseline characteristics of the chemotherapy-treated cohort are summarized in Supplementary Table S3. A total of 18,693 participants were included, with a mean age of 57.28 years (SD, 7.78); 67.1% were female, and the majority were White (95.7%). Most participants were never or former smokers (87.1%), and a high proportion reported current alcohol consumption (90.9%). Overall, baseline cardiometabolic and renal function indicators, including glucose, HbA1c, lipid fractions, inflammatory markers, and creatinine levels, were largely within normal ranges. Regarding cancer-related treatments, approximately half of the participants had documentation of chemotherapy sessions for neoplasm (50.3%), while 38.6% had a personal history of chemotherapy. Among cancer types, breast cancer was the most common malignancy (35.5%), followed by colorectal cancer (15.0%) and non-Hodgkin lymphoma (7.9%). Radiotherapy-related procedures and cardiovascular medication use were recorded in a minority of participants.

### Construction of the cardiovascular risk feature set

To identify stable features suitable for unsupervised clustering, we applied 10-fold cross-validated LASSO regression to 36 candidate variables encompassing sociodemographic characteristics, lifestyle factors, cardiometabolic and inflammatory biomarkers, renal function indicators, cancer-related characteristics, treatment exposures, and baseline medication use. These variables included age, sex, race, Townsend deprivation index, body mass index, physical activity, sleep duration, smoking status, alcohol intake, hypertension, cancer type, chemotherapy-related categories, radiotherapy-related procedures, glucose, HbA1c, creatinine, C-reactive protein, total cholesterol, HDL cholesterol, triglycerides, and use of lipid-lowering, antihypertensive, and antidiabetic medications (Supplementary Figure S1).

### Unsupervised clustering structure and subgroup identification

**Figure 2:**
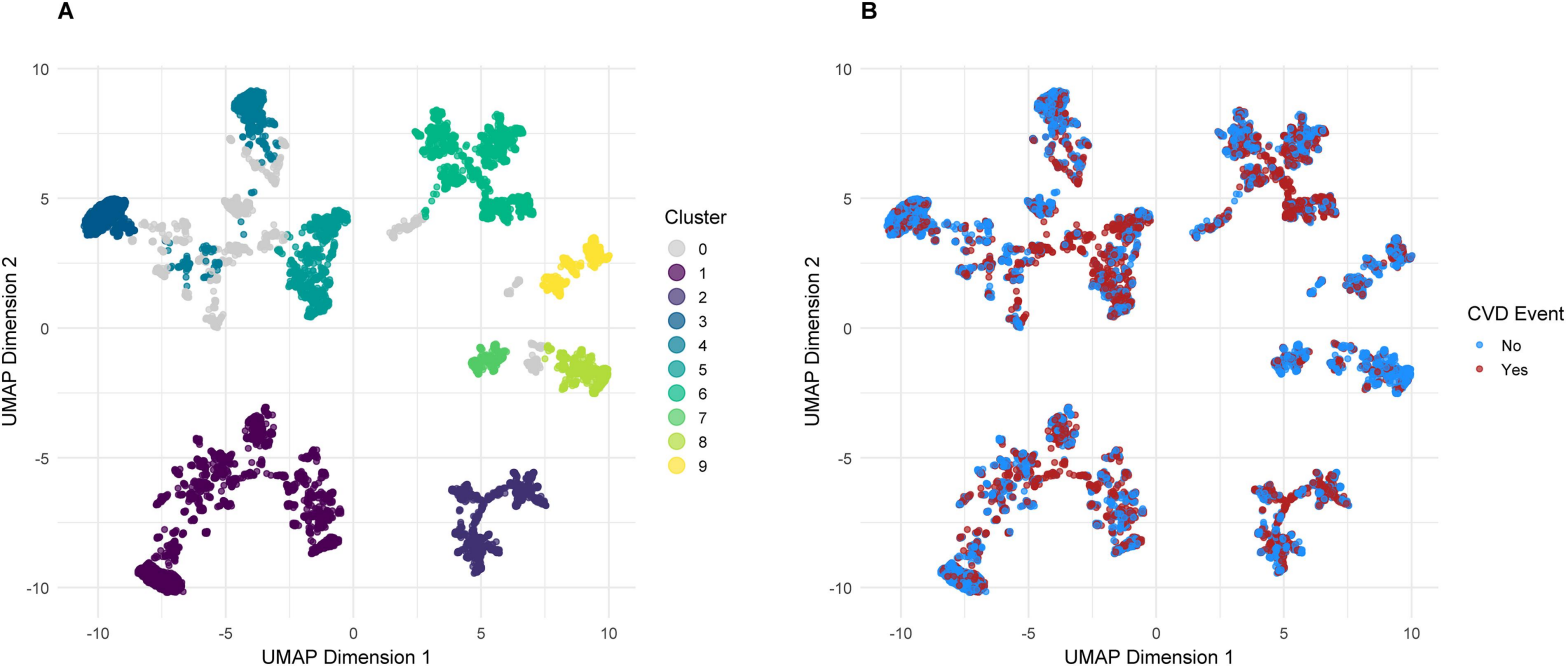
Visualization of patient clusters using UMAP analysis. **(A)** The distribution of samples colored by identified clusters. **(B)** The distribution of samples colored by CVD event status. Panel A shows a two-dimensional UMAP embedding of the study population based on selected baseline features, with points colored according to cluster assignment. Each point represents an individual participant, and clusters reflect groups identified by unsupervised clustering in the reduced feature space. Panel B displays the same UMAP embedding, with points colored according to the occurrence of CVD events during follow-up. UMAP, Uniform Manifold Approximation and Projection; CVD, cardiovascular disease.

Based on the selected features, the clustering achieved a mean silhouette coefficient of 0.633, a Calinski-Harabasz index of 29122.9, and a Dunn index of 0.0226, resulting in the identification of 9 major clusters, with 13.8% of observations classified as noise and excluded from downstream analyses (Figure 2).

Based on chemotherapy exposure patterns, the clusters could be broadly grouped into three categories. The first category consisted of clusters characterized by chemotherapy session for neoplasm, including Clusters 3, 4, 5, and 6. All individuals in these clusters had records of chemotherapy sessions for neoplasm. Clusters 3, 4, and 5 were predominantly female, with a high proportion of breast cancer. In contrast, Cluster 6 was mainly composed of male patients, with a higher proportion of colorectal cancer. This cluster also showed higher age, BMI, and creatinine levels, lower HDL levels, and a higher use of antihypertensive and lipid-lowering medications. In Clusters 4 and 5, a higher proportion of participants had a history of smoking and radiotherapy exposure. Cluster 5 also had a higher prevalence of prior chemotherapy history.

The second category included clusters characterized by other chemotherapy, namely Clusters 1 and 2. In both clusters, all individuals had records of other chemotherapy, while chemotherapy sessions for neoplasm and prior chemotherapy history were uncommon. Cluster 1 was predominantly female, with a higher proportion of breast and gynecological cancers. Participants in this cluster had lower rates of smoking and alcohol consumption and lower creatinine levels. Cluster 2 was mainly male, with a higher proportion of prostate and colorectal cancers. This cluster was characterized by higher BMI and creatinine levels, lower HDL levels, and a higher use of antihypertensive, lipid-lowering, and antidiabetic medications, as well as a higher prevalence of smoking.

The third category consisted of clusters defined by a history of prior chemotherapy, including Clusters 7, 8, and 9. These clusters had no records of chemotherapy sessions for neoplasm or other chemotherapy but were characterized by prior chemotherapy exposure. Cluster 7 had a higher proportion of former smokers. Cluster 8 showed lower BMI, HbA1c, and smoking prevalence, along with higher HDL levels. Cluster 9 was predominantly male, with a higher proportion of prostate and colorectal cancers. It was also characterized by a higher proportion of current smokers, higher creatinine levels, lower HDL levels, and greater radiotherapy exposure.

Although Cluster 3 showed 100% current alcohol consumption, this variable had limited discriminatory value in this cohort, as most clusters had a high proportion of current drinkers. In contrast, Cluster 3 was better characterized by a more favorable cardiovascular risk profile, including minimal smoking, very low prevalence of baseline hypertension and cardiovascular medication use, higher HDL levels, and lower creatinine levels. Therefore, using Cluster 3 as the reference group was considered reasonable from both clinical and statistical perspectives. Complete definitions of baseline characteristics for all clusters are provided in Supplementary Table S4.

### Characteristics of noise-classified participants

To improve transparency, we compared baseline characteristics between the core clustered population and participants classified as noise by HDBSCAN (Supplementary Table S5). Overall, the noise group showed a distinct clinical profile, with a markedly lower proportion of male participants, a higher prevalence of current smoking, more frequent active chemotherapy-related records, greater radiotherapy exposure, and higher use of baseline antihypertensive medications than the core clustered population. These findings suggest that participants classified as noise represented a more heterogeneous subgroup with atypical combinations of clinical and treatment-related features.

### Evaluation of cluster robustness

We further evaluated cluster robustness using 200 bootstrap resamplings and comparisons with alternative clustering algorithms. In the bootstrap analysis, Jaccard similarity between different clusters remained uniformly low (<0.001). Clustering results were highly concordant with those obtained using K-means, hierarchical clustering, and partitioning around medoids, with a mean ARI of 0.71 and a mean NMI of 0.84 **(**Supplementary Tables S6, S7 and Supplementary Figure S2).

### SHAP interpretability analysis

**Figure 3:**
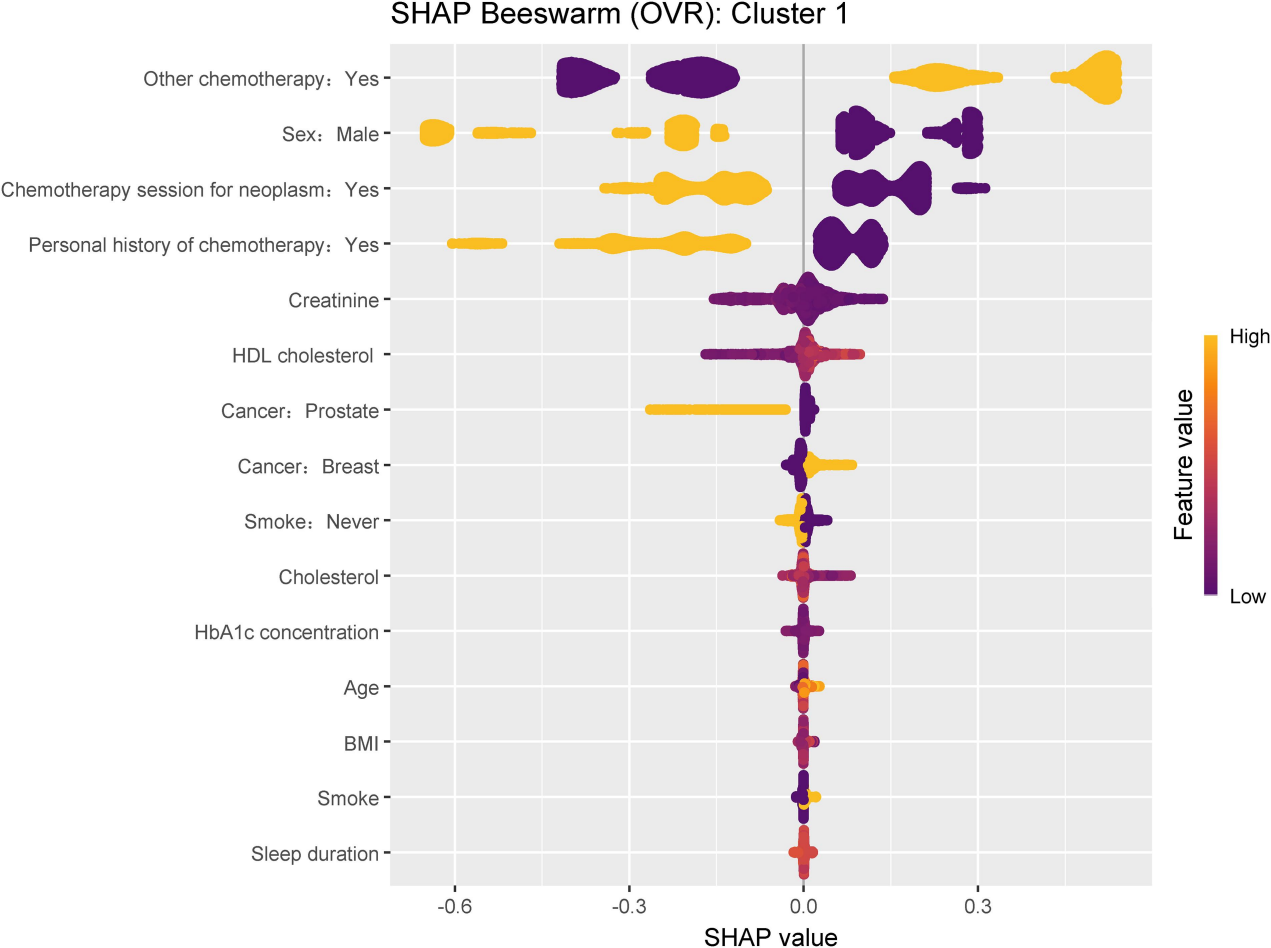
Top predictive features for Cluster 1 identified by SHAP analysis. SHAP beeswarm plots displaying the most significant features driving the classification of Cluster 1. Each point represents an individual observation. The horizontal position indicates the SHAP value, reflecting the direction and magnitude of a given feature's contribution to the model prediction for the corresponding cluster. Positive SHAP values indicate a higher predicted probability of belonging to the target cluster, whereas negative values indicate a lower predicted probability. Features are displayed on the y-axis in descending order of importance, defined by the mean absolute SHAP value. Point color denotes the original feature value, ranging from low (purple) to high (yellow). SHAP, SHapley Additive exPlanations; OVR, One-vs-Rest; NEC, not elsewhere classified; HbA1c, haemoglobin A1c; HDL, high-density lipoprotein; BMI, body mass index; TDI, Townsend deprivation index.

The LightGBM confusion matrix demonstrates low misclassification rates (Supplementary Figure S3). LightGBM-based SHAP analysis showed that feature contribution rankings in a subset of clusters were primarily driven by chemotherapy-related variables (Figure 3). Overall, chemotherapy-related variables ranked among the most important contributors across multiple clusters. In particular, receipt of chemotherapy sessions for neoplasm, other chemotherapy exposure, and history of chemotherapy were repeatedly identified as leading features. In Clusters 3–6, receipt of chemotherapy sessions for neoplasm was one of the major contributing variables. In Clusters 5 and 6, this pattern was accompanied by relatively high contributions from history of chemotherapy or other chemotherapy exposure. In Clusters 1 and 2, other chemotherapy exposure ranked among the top contributing features, while sex also showed a strong contribution. Notably, male sex was the single most important feature in Cluster 2. In Clusters 7–9, history of chemotherapy consistently showed high contribution across several clusters, and receipt of chemotherapy sessions for neoplasm as well as other chemotherapy exposure also appeared among the leading features. In addition to chemotherapy-related variables, sex, smoking status, creatinine, and HDL cholesterol also made important contributions in some clusters, jointly shaping the distinguishing profiles of these subgroups. Complete SHAP rankings for all clusters are provided in Supplementary Table S8 and Supplementary Figure S4.

### Heterogeneity of cardiovascular outcomes across clusters

Using Cluster 3 as the reference group, a clear gradient of cardiovascular risk was observed across the 9 clusters.

#### Risk differences in overall cardiovascular events

**Figure 4:**
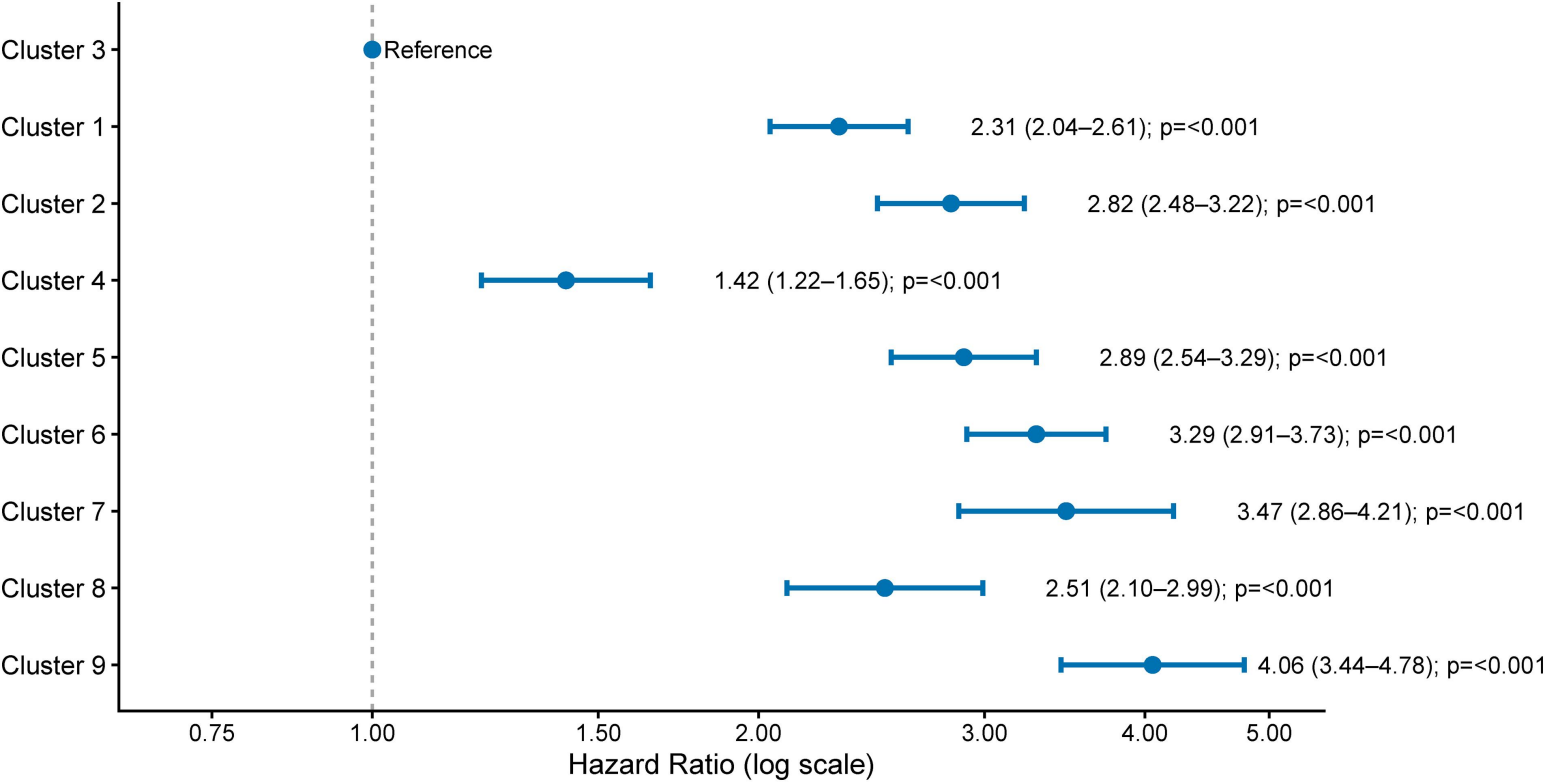
Cox regression for CVD across clusters. Hazard ratios (HRs) and 95% confidence intervals (CIs) for the study outcome according to cluster membership are shown, with Cluster 3 serving as the reference group. Estimates were derived from Cox proportional hazards regression models. Points indicate HRs, and horizontal bars represent 95% CIs. The vertical dashed line denotes an HR of 1. Values to the right of the plot report the HRs with corresponding 95% CIs and P values. The x-axis is presented on a logarithmic scale.

Across the 9 clusters, the risk of composite CVD differed substantially (Figure 4). Using Cluster 3 as the reference group, most clusters showed an increased risk of overall CVD. Cluster 9 had the highest risk [HR, 4.06 (95% CI, 3.44–4.78)], followed by Cluster 7 [HR, 3.47 (95% CI, 2.86–4.21)] and Cluster 6 [HR, 3.29 (95% CI, 2.91–3.73)].

Clusters 2 and 5 also showed elevated risks [HR, 2.82 [95% CI, 2.48–3.22] and 2.89 [95% CI, 2.54–3.29], respectively], while Clusters 1 and 8 had risks above the reference group as well. In contrast, Cluster 4 showed a more modest increase in risk [HR, 1.42 (95% CI, 1.22–1.65)].

#### Risk differences across specific cardiovascular disease subtypes

In analyses of specific CVD outcomes, substantial risk heterogeneity was also observed across clusters. In the analysis of hypertension, an increased risk was observed across multiple clusters, with Cluster 2 showing the highest risk [HR, 3.65 (95% CI, 3.07–4.34)], while no clear association was observed for Cluster 8.

For ischemic heart disease–related outcomes, including angina, acute myocardial infarction, and ischemic heart disease, Clusters 2 and 6 consistently showed higher risks. For example, for angina, the HRs were 7.63 (95% CI, 4.29–13.59) for Cluster 2 and 4.41 (95% CI, 2.50–7.80) for Cluster 6. For acute myocardial infarction, the corresponding HRs were 7.46 (95% CI, 3.86–14.43) and 5.49 (95% CI, 2.88–10.45). For ischemic heart disease, Cluster 2 showed an HR of 7.97 (95% CI, 5.44–11.66), and Cluster 6 showed an HR of 5.26 (95% CI, 3.62–7.64).

For arrhythmia and thromboembolic outcomes, risk differences were also evident. For atrial fibrillation, Cluster 9 showed the highest risk [HR, 4.67 (95% CI, 3.04–7.17)], while Clusters 6 and 2 also demonstrated elevated risks. For pulmonary embolism and ischemic stroke, increased risks were observed in most clusters. Notably, Cluster 7 showed an HR of 3.94 (95% CI, 2.48–6.27) for pulmonary embolism, and Cluster 9 showed an HR of 5.40 (95% CI, 2.58–11.31) for ischemic stroke.

In contrast, no clear differences were observed across clusters for hemorrhagic stroke. For aortic aneurysm and dissection, the confidence intervals were wide, indicating limited precision of the estimates. Complete Cox regression results for all specific outcomes are provided in Supplementary Table S9.

### Incremental predictive value of cluster information in cardiovascular disease risk models

**Figure 5:**
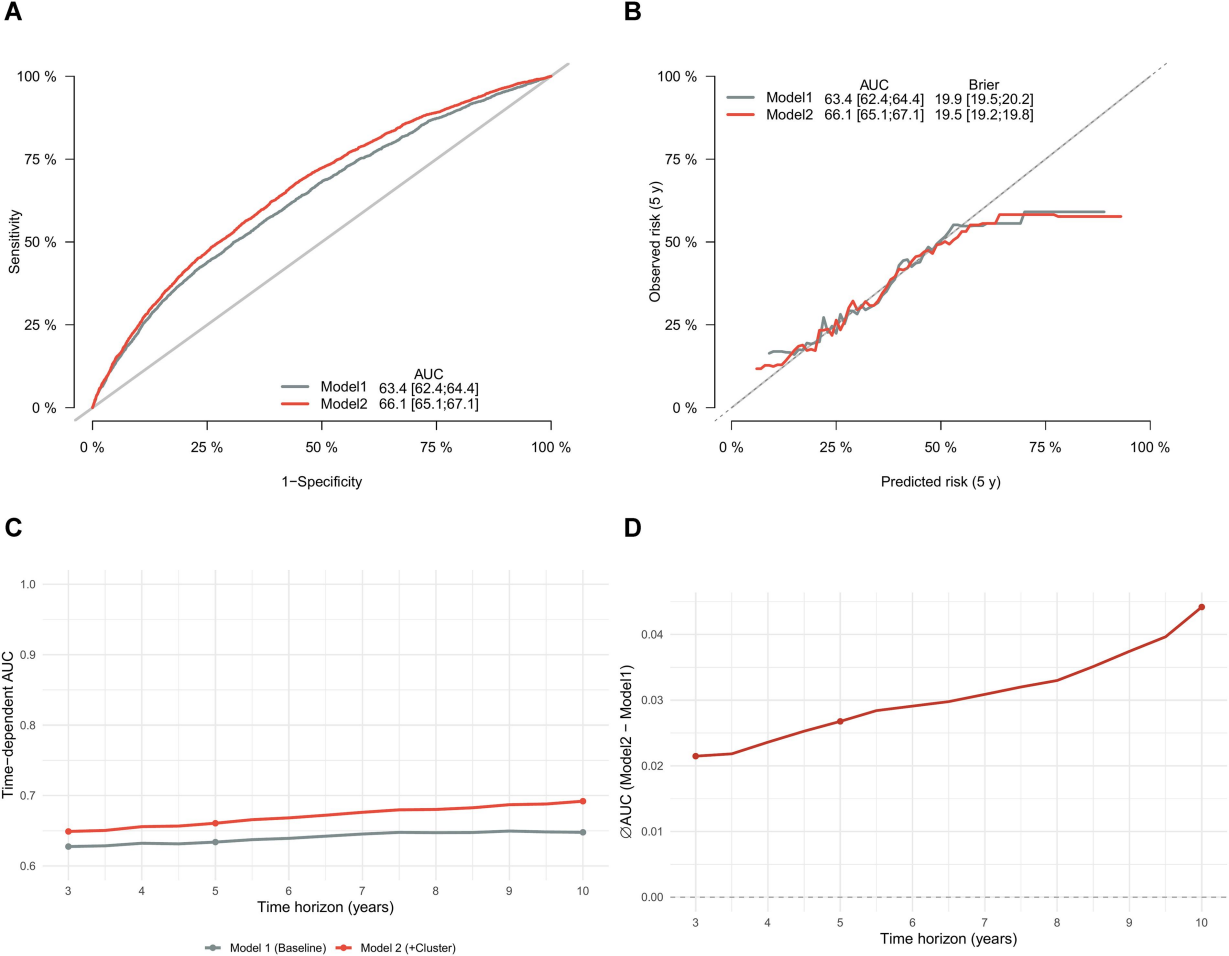
Comparison of predictive performance between the baseline and cluster-enhanced models. **(A)** Five-year receiver operating characteristic (ROC) curves comparing the baseline model (Model 1) and the cluster-enhanced model (Model 2). **(B)** Five-year calibration plots assessing the agreement between predicted and observed cardiovascular disease risks. **(C)** Time-dependent AUC trajectories for both models across a 3-to-10-year time horizon. **(D)** Difference in AUC between Model 2 and Model 1 (Model 2 − Model 1) over time. AUC, area under the curve; ROC, receiver operating characteristic.

At the 10-year prediction time point, adding cluster membership to the baseline model improved discrimination, with the AUC increasing from 0.648 to 0.692 (ΔAUC = 0.044; *P* < 0.001). Calibration analysis showed a lower Brier score for Model 2 compared with Model 1. Time-dependent AUC analyses further demonstrated that Model 2 consistently outperformed Model 1 over the 3–10 year follow-up period (ΔAUC, 0.021–0.044) (Figure 5). In addition, Harrell's C-index increased from 0.611 to 0.625, with detailed numerical results provided in Supplementary Tables S10–S12.

### Sensitivity analyses

To evaluate the robustness of the findings, additional sensitivity analyses were performed. After excluding sex from the clustering features, HDBSCAN identified six core clusters and 862 noise points, and clear gradients in cardiovascular risk across clusters remained evident (Supplementary Figures S5, S6 and Supplementary Tables S13, S14).

In sex-stratified analyses, nine clusters were identified in women and three clusters in men. In both subgroups, heterogeneous patterns of cardiovascular risk across clusters were consistently observed, although the number and granularity of clusters differed between sexes (Supplementary Figures S7–S10 and Supplementary Tables S15–S18). Overall, the patterns of risk stratification were consistent with those observed in the primary analysis.

## Discussion

In this UK Biobank analysis of 18,693 chemotherapy-treated cancer patients without a prior history of CVD, a clear gradient in overall cardiovascular risk was observed. Unsupervised clustering integrating demographic characteristics, metabolic status and renal function indicators, lifestyle factors, and treatment-related information identified 9 subgroups with pronounced heterogeneity in baseline risk composition and incident cardiovascular outcomes. Relative to a reference subgroup characterized by lower metabolic burden, preserved renal function, less radiotherapy exposure and healthier lifestyle factors (Cluster 3), cardiovascular risk increased in a stepwise manner across clusters, from lower-risk groups (Cluster 4; HRs, approximately 1.42; Cluster 1; HRs, approximately 2.31), through intermediate-risk to higher-risk clusters (Clusters 2, 5, 6, and 8), to an extreme-risk cluster (Cluster 7; HRs, approximately 3.47; Cluster 9; HRs, approximately 4.06). These findings extend prior evidence of elevated overall CVD risk among cancer patients by demonstrating substantial cardio-oncology risk heterogeneity within populations already exposed to chemotherapy.

In terms of phenotypic composition, the differences in overall CVD risk across subgroups largely correspond to chemotherapy-related clinical trajectories. The reference group, Cluster 3, and the relatively low-risk Cluster 4 are primarily characterised by records of chemotherapy for malignant tumours, whereas higher-risk subgroups, particularly Clusters 7 and 9, are predominantly defined by a phenotype dominated by a history of chemotherapy; At the same time, the intermediate-risk subgroups exhibit mixed characteristics involving other chemotherapy-related records or more complex treatment backgrounds. We believe that this pattern should not be simply interpreted as reflecting direct differences in the toxicity of different chemotherapy regimens; rather, it is more likely related to differences in cumulative treatment exposure and follow-up duration (18, 31, 32). The Danish National Lymphoma Cohort Study demonstrated that, compared with non-anthracycline regimens, anthracycline-containing regimens were associated with a higher risk of CHF, with the risk being most pronounced in patients receiving >6 cycles of R-CHOP/CHOEP, suggesting that cumulative anthracycline exposure may be a significant contributor to differences in long-term cardiovascular risk (31). In addition, a meta-analysis including 53 studies and 35,651 patients showed that the risk of chemotherapy-related cardiac dysfunction was highest during the first 6 months after chemotherapy, then gradually decreased, and was no longer statistically significant after 6 years (33). A cardio-oncology review also noted that most CTR-CVT events occur within 1 year after treatment completion. Late cardiotoxicity is less common, but it can still appear more than 1 year after treatment, or even much later (18, 32). Together, these findings suggest that the cardiovascular effects of chemotherapy may be strongly time-dependent (18, 32, 33).

Beyond treatment trajectories, we also observed that certain subgroups exhibited a highly homogeneous gender composition. This pattern was partly consistent with the cancer distribution in our cohort. Given the high prevalence of breast cancer in the cohort and its inherent significant female bias, there is a natural correlation between gender and cancer type composition (34). In this context, gender may not only directly influence the clustering structure but may also indirectly contribute to subgroup classification through its association with cancer type, treatment pathways, and baseline risk burden (35, 36). Sensitivity analyses further indicate that, after removing sex from the clustering variables, the overall cluster structure and the corresponding gradient in cardiovascular risk were largely preserved. Likewise, when clustering was performed separately within women and men, risk heterogeneity still remained. This suggests that although gender exerts a significant influence on the formation of the clustering structure, it is insufficient on its own to explain the risk stratification between different subgroups.

Further analysis of baseline characteristics across subgroups reveals systematic differences between subgroups in smoking status, HDL cholesterol levels, renal function indicators, baseline hypertension and the use of related medications. These are all established markers of adverse cardiovascular risk (34, 37–40). From this perspective, the subgroups identified in this study are better understood as multidimensional cardio-oncology risk profiles rather than ‘cardiovascular phenotypes' in the traditional sense. These risk structures encompass not only treatment-related information but also multifaceted factors such as demographic characteristics, lifestyle factors, and metabolic status. Together, they shape the heterogeneity of long-term cardiovascular risk in cancer patients following chemotherapy and suggest that, within this population, stratification based solely on cancer type, individual treatment records, or traditional risk factors may be insufficient to adequately identify those at genuinely high risk.

Current cardio-oncology consensus statements emphasize baseline cardiovascular risk assessment before initiation of systemic anticancer therapy, followed by risk-adapted surveillance during follow-up. However, a clear gap remains in the development and validation of pragmatic and scalable risk stratification tools applicable to real-world cancer populations (14). Given the growing burden of cardiovascular morbidity among cancer patients, cardiovascular risk assessment and management should be prioritized in this population. Although multiple large-scale cohort studies and systematic reviews have consistently shown higher cardiovascular risk among cancer patients than among the general population, refined risk phenotyping strategies tailored to cancer patients remain limited (41). In this context, our findings align with recommendations from the ESC Cardio-Oncology Working Group, as articulated by Lyon and colleagues, emphasizing the need for interpretable and individualized cardiovascular risk stratification models (37). Early identification of cancer patients at high risk for cardiovascular complications is critical. Future studies should focus on external validation and refinement of such approaches, incorporating dynamic weighting of established and emerging risk factors and evaluating their associations with overall survival and cardiovascular-related and cancer-related outcomes.

In predictive performance analyses, addition of cluster membership to baseline models incorporating demographic characteristics, metabolic status, renal function indicators, lifestyle factors, and chemotherapy exposures resulted in statistically significant but modest improvements in model discrimination. This pattern is consistent with prior risk modeling studies (42, 43) in which the incremental gain in the area under the receiver operating characteristic curve is often limited (44) when new predictors are added to baseline models with moderate discriminatory performance. In a UK Biobank analysis, incorporation of a polygenic risk score into the Pooled Cohort Equations increased the C statistic by approximately 0.02 while yielding a net reclassification improvement of 4.4% (45). Such modest but directionally consistent gains are well recognized in risk prediction research when novel predictors are added to baseline models with moderate discrimination. Similar patterns have been reported in phenomapping studies of heart failure with preserved ejection fraction (46) and in investigations of obesity-related metabolic subtypes (47), in which phenotype labels confer limited improvement in C statistics but provide particular value for identifying well-defined high-risk subgroups and informing trial stratification and tailored follow-up strategies. From a clinical perspective, these findings may provide preliminary insights into how clustering approaches could be explored for risk stratification in cardio-oncology populations. However, given the modest incremental predictive improvement, further validation and refinement are required before such approaches can be considered for clinical application. Such approaches could facilitate pragmatic risk stratification, enabling more intensive metabolic and blood pressure management and closer cardiovascular monitoring in high-risk phenotypes, while avoiding unnecessary interventions in protective phenotypes, thereby supporting more efficient long-term cardiovascular care in resource-constrained settings.

### Strengths and limitations

This study has several strengths. It was conducted within the UK Biobank, a large prospective real-world cohort, and focused on chemotherapy-treated cancer patients without preexisting CVD, yielding a well-characterized population with complete follow-up and standardized outcome definitions. Rather than limiting the analysis to a single cancer type or chemotherapeutic agent, we integrated demographic, lifestyle, metabolic, renal, and treatment-related factors and applied unsupervised machine learning to identify distinct cardio-oncology phenotypes. These phenotypes showed a consistent gradient in both overall CVD risk and multiple specific cardiovascular outcomes. Furthermore, after incorporating cluster membership into the conventional baseline model, both discrimination and calibration improved. Although the increase in AUC was relatively modest, the improvement was generally consistent across different evaluation metrics, suggesting that this stratification approach may provide incremental value for risk identification.

However, several limitations of this study should be acknowledged. First, participants in the UK Biobank are predominantly middle-aged and older individuals of European ancestry, and differences in chemotherapy practice patterns and underlying risk profiles across populations may limit the generalizability of the identified phenotypes to other populations and health care systems. In addition, some clusters showed marked sex imbalance, which was partly related to the underlying distribution of cancer types, particularly the predominance of breast cancer among female participants. Therefore, the identified phenotypes may reflect not only cardiovascular risk characteristics but also the joint influence of sex, cancer type, and treatment patterns. This underscores the need for further validation in independent, multicenter prospective cardio-oncology cohorts. Second, although multiple imputation was adopted in the revised analysis to reduce sample loss associated with complete-case analysis, this approach still relies on the missing-at-random assumption; if missingness was related to unmeasured factors, residual bias may remain. Third, follow-up in this study started from the date of the first chemotherapy record, whereas some covariates were obtained from baseline assessment. Therefore, age and other baseline characteristics were not perfectly aligned with the time of chemotherapy initiation, particularly for participants with a long interval between cohort enrollment and treatment, which may have introduced some degree of bias in risk characterization. Finally, although radiotherapy and selected baseline cardiovascular medications were additionally incorporated in the revised analysis, the database still lacked more detailed information on tumor stage, specific chemotherapy regimens, routes of administration, and cumulative doses. Therefore, despite the use of LASSO for feature selection and multivariable adjustment, residual confounding, including confounding by indication, may still remain, and causal interpretation of the findings should be made with caution.

## Conclusion

In this large real-world cohort, substantial heterogeneity in cardiovascular risk was identified among cancer patients treated with chemotherapy, with distinct cardio-oncology risk phenotypes characterized by differences in metabolic burden, renal function, lifestyle factors, and treatment exposure patterns. These findings suggest that phenotype-based profiling may provide a useful exploratory framework for understanding risk heterogeneity and refining risk stratification in cardio-oncology populations. However, given the modest incremental predictive improvement observed after adding phenotype labels, these results should be interpreted cautiously. Further validation in independent prospective cohorts, together with incorporation of more granular treatment information and longitudinal biomarkers, is needed before clinical implementation.

## Data Availability

The dataset analyzed in this study was obtained from the UK Biobank under a specific license and cannot be shared by the authors due to these licensing restrictions. Interested researchers can apply for access directly through the UK Biobank. Requests regarding the study's methodology or analysis code can be directed to the corresponding authors.
