# Supplementary material for: Cardio-Oncology Risk Phenotypes in Cancer Patients Treated with Chemotherapy: A Data-Driven Analysis
Source: Front Cardiovasc Med. 2026 Aug 3;13:1826258. doi: 10.3389/fcvm.2026.1826258 (PMC13478683; doi:10.3389/fcvm.2026.1826258)
Supplement: Supplementary file 1 [file Datasheet1.docx]

**Supplementary Material**

**Supplementary Tables**

**Supplementary Table S1. Variable definitions and corresponding UK Biobank field IDs**

**Supplementary Table S2. Variable definitions and corresponding UK Biobank field IDs for medication covariates.**

**Supplementary Table S3. Baseline characteristics involved in the reaserch**

**Supplementary Table S4. Cluster variables based on UMAP-HDBSAN**

**Supplementary Table S5. Baseline characteristics of noise group compared to the core clustered population.**

**Supplementary Table S6. Bootstrap sensitivity analysis of cluster pairs based on Jaccard Index**

**Supplementary Table S7. Algorithm sensitivity analysis using NMI and ARI**

**Supplementary Table S8. Top 10 SHAP features for cluster identification**

**Supplementary Table S9. Cox regression analysis of CVD risk across clusters**

**Supplementary Table S10. Harrell’s C-index for predictied models**

**Supplementary Table S11. Model performance evaluation for CVD risk prediction at 3,5 and 10 years**

**Supplementary Table S12. Time-dependent AUC for CVD risk prediction models**

**Supplementary Table S13. Baseline characteristics of identified clusters after excluding sex from the clustering input variables
Supplementary Table S14. Hazard ratios for specific cardiovascular outcomes across clusters after excluding sex from the clustering input variables**

**Supplementary Table S15. Baseline characteristics of identified clusters in the female subgroup**

**Supplementary Table S16. Hazard ratios for specific cardiovascular outcomes across clusters in the female subgroup**

**Supplementary Table S17. Baseline characteristics of identified clusters in the male subgroup**

**Supplementary Table S18. Hazard ratios for specific cardiovascular outcomes across clusters in the male subgroup**

**Supplementary Figures**

**Supplementary Figure S1. Lasso coefficient paths and cross-validation curve**

**Supplementary Figure S2. Algorithmic clustering sensitivity analysis**

**Supplementary Figure S3. LightGBM confusion matrix for cluster classification**

**Supplementary Figure S4. Top predictive features for each patient cluster identified by SHAP analysis**

**Supplementary Figure S5. UMAP clustering and cardiovascular event distribution after excluding sex from the clustering input variables
Supplementary Figure S6. Forest plot of hazard ratios for overall cardiovascular disease risk across clusters after excluding sex from the clustering input variables**

**Supplementary Figure S7. UMAP clustering and cardiovascular event distribution in the female subgroup**

**Supplementary Figure S8. Forest plot of hazard ratios for overall cardiovascular disease risk across clusters in the female subgroup**

**Supplementary Figure S9. UMAP clustering and cardiovascular event distribution in the male subgroup**

**Supplementary Figure S10. Forest plot of hazard ratios for overall cardiovascular disease risk across clusters in the male subgroup**

**Supplementary Table S1. Variable definitions and corresponding UK Biobank field IDs**

| **Category** | **Variable name** | **UK Biobank Field ID** | **Code** |
| --- | --- | --- | --- |
| CVD | Ischemic heart diseases (IHD) | 41270 | I20、I21、I22、I23、I24、I25 |
|  | Hemorrhagic stroke |  | I60、I61、I62 |
|  | Ischemic stroke |  | I63、I64、I65、I66 |
|  | Essential (primary) hypertension |  | I10 |
|  | Angina pectoris |  | I20 |
|  | Acute myocardial infarction |  | I21 |
|  | Pulmonary embolism |  | I26 |
|  | Atrial fibrillation and flutter |  | I48 |
|  | Aortic aneurysm and dissection |  | I71 |
| Chemotherapy | Follow-up examination after chemotherapy for malignant neoplasm |  | Z08.2 |
|  | Chemotherapy session for neoplasm |  | Z51.1 |
|  | Other chemotherapy |  | Z51.2 |
|  | Convalescence following chemotherapy |  | Z54.2 |
|  | Personal history of chemotherapy for neoplastic disease |  | Z92.6 |
| Cancer | Thyroid |  | C73 |
|  | Esophagus |  | C15 |
|  | Lung |  | C34 |
|  | Stomach |  | C16 |
|  | Liver |  | C22 |
|  | Pancreas |  | C25 |
|  | Colorectum |  | C18 - C20 |
|  | Small intestine |  | C17 |
|  | Ureter |  | C66 |
|  | Renal |  | C64 - C65 |
|  | Bladder |  | C67 |
|  | Prostate |  | C61 |
|  | Penis |  | C60 |
|  | Cervix |  | C53 |
|  | Ovary |  | C56 |
|  | Vulva |  | C51 |
|  | Anal canal |  | C21 |
|  | Bone |  | C40 - C41 |
|  | Skin |  | C43 - C44 |
|  | Non-Hodgkin |  | C82 - C85 |
|  | Hodgkin |  | C81 |
|  | Leukemia |  | C91 - C95 |
| Radiotherapy | Delivery of a fraction of intracavitary radiotherapy | 41270 | X65.2 |
|  | Delivery of a fraction of interstitial radiotherapy |  | X65.3 |
|  | Delivery of a fraction of external beam radiotherapy NEC |  | X65.4 |
|  | Oral delivery of radiotherapy for thyroid ablation |  | X65.5 |
|  | Other specified radiotherapy delivery |  | X65.8 |
|  | Unspecified radiotherapy delivery |  | X65.9 |
|  | Preparation for simple radiotherapy with imaging and dosimetry |  | X67.4 |
|  | Preparation for simple radiotherapy with imaging and simple calculation |  | X67.5 |
|  | Preparation for superficial radiotherapy with simple calculation |  | X67.6 |
|  | Preparation for complex conformal radiotherapy |  | X67.7 |
|  | Unspecified preparation for external beam radiotherapy |  | X67.9 |
|  | Megavoltage treatment for simple radiotherapy |  | Y91.2 |
|  | Superficial or orthovoltage treatment for radiotherapy |  | Y91.3 |
|  | Megavoltage treatment for adaptive radiotherapy |  | Y91.4 |
|  | Megavoltage treatment for hypofractionated stereotactic radiotherapy |  | Y91.5 |
|  | Unspecified external beam radiotherapy |  | Y91.9 |
|  | Technical support for preparation for radiotherapy |  | Y92.1 |
|  | Other specified support for preparation for radiotherapy |  | Y92.8 |
|  | Unspecified support for preparation for radiotherapy |  | Y92.9 |
| Anthropometric indicators | Body mass index (BMI) | 21001 | - |
| Sociodemographic and lifestyle | Age | 21022 | - |
|  | Sex | 31 | - |
|  | Smoking status | 20116 | - |
|  | Alcohol intake frequency (current) | 1558 | - |
|  | Townsend deprivation index | 22189 | - |
|  | Race | 21000 | - |
|  | MET minutes per week for all activity | [22040](https://biobank.ndph.ox.ac.uk/showcase/field.cgi?id=22040) | - |
|  | Sleep duration | 1160 | - |
| Metabolic biomarkers | Glucose | 23470 | - |
|  | [Glycated haemoglobin (HbA1c)](https://biobank.ndph.ox.ac.uk/showcase/field.cgi?id=30750) | 30750 | - |
|  | Creatinine | 30700 | - |
|  | Cholesterol | 30690 | - |
|  | C-reactive protein (CRP) | [30712](https://biobank.ndph.ox.ac.uk/ukb/field.cgi?id=30712) | - |
|  | HDL Cholesterol | 30760 | - |
|  | Triglycerides | 30870 | - |

Abbreviations: CVD, cardiovascular diseases; MET, metabolic equivalent of task; HDL, High-density lipoprotein.

**Supplementary Table S2. Variable definitions and corresponding UK Biobank field IDs for medication covariates.**

| Medication covariates | UK Biobank Field ID |
| --- | --- |
| Lipid-lowering medication | 20003;6177;6153 |
| Lipid Lowering Drug; Atorvastatin; Velastatin; Rosuvastatin; Simvastatin; Fluvastatin; pravastatin;Eptastatin; lipostat 10mg tablet; Atromid-s 500mg capsule; Modalim 100mg Tablet; crestor 10mg tablet; Bezafibrate; Fenofibrate; Bezafibrate product; Clofibrate; Gemfibrozil; Gemfibrozil product; bezalip; bezalip-mono 400mg m/r tablet; questran 4g/sachet powder; cholestyramine+aspartame 4g/sachet powder; lopid 300 capsule; colestipol; synvinolin; Acipimox; Nicotinic Acid Product; probucol;nicofuranose; lurselle 250mg tablet; colestid 5g/sachet granules; olbetam 250mg capsule; cholestyramine; colestyramine; cholestyramine product;  1140861922; 1141146234; 1140910654; 1141192410; 1140861958; 1140888594; 1140888648; 1140861970; 1140910632; 140861946;1140862028; 1141192414; 1140861924; 1140861954; 1141157260; 1140861944; 1140861856; 1141157262; 1140861926; 1140861928; 1140861936; 1140861942; 140861858;1140888590;1140910652;1140861868;1140861892;1140861876; 1140861866; 1140861848; 1140861878;1140861894; 1140865576;1140909780; 1141157416; | |
| Antihypertensive medication | 20003;6177;6153 |
| capozide 50mg tablets x28; lisinopril ; carace 2.5mg tablet; zestril 2.5mg tablet; quinapril; accuretic tablet; captopril; acepril 12.5mg tablet; capoten 12.5mg tablet; captopril+hydrochlorothiazide 25mg/12.5mg tablet; innovace 2.5mg tablet; innozide tablet; enalapril maleate+hydrochlorothiazide 20mg/12.5mg tablet; coversyl 2mg tablet; ramipril; staril 10mg tablet; cilazapril; vascace 250micrograms tablet; trandolapril; gopten 500 micrograms capsule; odrik 500micrograms capsule; zestoretic 10 tablet; carace 10 plus tablet; lisinopril+hydrochlorothiazide 10mg/12.5mg tablet; indapamide; amlodipine; accupro 5mg tablet; enalapril; fosinopril; perindopril; felodipine; losartan; cozaar half strength 25mg tablet; perdix 7.5mg tablet; valsartan; diovan 40mg capsule; ecopace 12.5mg tablet; kaplon 12.5mg tablet; losartan potassium+hydrochlorothiazide 10mg/12.5mg tablet; cozaar-comp 50mg/12.5mg tablet; irbesartan; aprovel 75mg tablet; tarka 2mg/180mg m/r capsule; trandolapril+verapamil hydrochloride; candesartan cilexetil; amias 2mg tablet; imidapril hydrochloride; tanatril 5mg tablet; felodipine+ramipril; triapin mite 2.5mg/2.5mg tablet; telmisartan; hyteneze 12.5 tablet; tensopril 12.5mg tablet; capto-co 25mg/12.5mg tablet; pralenal 2.5mg tablet; eprosartan; micardis 20mg tablet; irbesartan+hydrochlorothiazide 150mg/12.5mg tablet; coaprovel 150mg/12.5mg tablet; cozaar 25mg tablet; perindopril+indapamide; coversyl plus 4mg/1.25mg tablet; telmisartan+hydrochlorothiazide 40mg/12.5mg tablet; tritace 1.25mg tablet; caralpha 10/12.5mg tablet; Olmesartan; olmetec 10mg tablet; lopace 2.5mg capsule; ranace 1.25mg capsule; lisicostad hct 10/12.5mg tablet; valsartan+hydrochlorothiazide 80mg/12.5mg tablet; co-diovan 80mg/12.5mg tablet; centyl 2.5mg tablet; urizide 5mg tablet; enduron 5mg tablet; esidrex k tablet; hygroton k tablet combination pack; navidrex-k tablet; serpasil-esidrex tablet; sotalol hydrochloride+hydrochlorothiazide 80mg/12.5mg tablet; metoprolol tartrate+hydrochlorothiazide 100mg/12.5mg tablet; acebutolol+hydrochlorothiazide 200mg/12.5mg tablet; methyldopa+hydrochlorothiazide 250mg/15mg tablet; quinalapril+hydrochlorothiazide 10mg/12.5mg tablet; bisoprolol fumarate+hydrochlorothiazide 10mg/6.25mg tablet; hydroflumethiazide; hydrenox 50mg tablet; mefruside; baycaron 25mg tablet; metolazone; xuret 500micrograms tablet; xipamide; diurexan 20mg tablet; aprinox 2.5mg tablet; berkozide 2.5mg tablet; neo-naclex 5mg tablet; chlorothiazide; saluric 500mg tablet; hygroton 50mg tablet; cyclopenthiazide; navidrex 500mcg tablet; hydrochlorothiazide; esidrex 25mg tablet; hydrosaluric 25mg tablet; amiloride hcl+cyclopenthiazide 2.5mg/250micrograms tablet; centyl k m/r tablet; neo-naclex k m/r tablet; neo-bendromax 2.5mg tablet; nindaxa 2.5mg tablet; chlortalidone; natramid 2.5mg tablet; opumide 2.5mg tablet; diltiazem hcl+hydrochlorothiazide 150mg/12.5mg m/r capsule; natrilix sr 1.5mg m/r tablet; triamterene+chlortalidone 50mg/50mg tablet; atenolol+chlortalidone; diuril 250mg/5ml oral suspension; Bendroflumethiazide; bendroflumethiazide+potassium 2.5mg/7.7mmol m/r tablet; nadolol+bendroflumethiazide 40mg/5mg tablet; timolol maleate+bendroflumethiazide 10mg/2.5mg tablet; atenolol+Bendroflumethiazide; brinaldix k tablet; diatensec 50mg tablet; laractone 25mg tablet; normetic tablet; synuretic tablet; vasetic co-amilozide 5/50mg tablet; pindolol; pindolol+clopamide 10mg/5mg tablet; timolol maleate+co-amilozide 10mg/2.5mg/25mg tablet; aridil 2.5mg/20mg tablet; spiro-co 25mg tablet; midamor 5mg tablet; berkamil 5mg tablet; spiroctan-m 200mg/10ml injection; spironolactone; aldactone 25mg tablet; lasix 20mg tablet; bumetanide; burinex 1mg tablet; spirospare 25mg tablet; spiretic 25mg tablet; spiroctan 25mg tablet; spirolone 25mg tablet; triamterene+benzthiazide 50mg/25mg capsule; triam-co tablet; triamterene+chlorthalidone 50mg/50mg tablet; triamterene+frusemide 50mg/40mg tablet; lasoride tablet; delvas tablet; navispare tablet; amilmaxco 5/50 tablet; burinex a tablet; triamaxco tablet; triamterene; dytac 50mg capsule; aldactide 25 tablet; amil-co tablet; dyazide tablet; dytide capsule; frumil tablet; kalspare tablet; moduret 25 tablet; fru-co tablet; moduretic tablet; amiloride hydrochloride+bumetanide 5mg/1mg tablet; burinex k m/r tablet; diumide-k continus m/r tablet; lasikal m/r tablet; bumetanide+potassium 500micrograms/7.7mmol m/r tablet; diuretic; amiloride; furosemide; amipramizide; zida-co 5mg/50mg tablet; co-triamterzide; co-amilozide; co-flumactone; co-amilofruse; amilamont 5mg/ml s/f oral solution; frusemek 5mg/40mg tablet; atenolol+co-amilozide; angiotensin ii receptor antagonist+diuretic; froop co 5mg/40mg tablet; komil 5/40 tablet; triamterene+furosemide 50mg/40mg tablet; furosemide+potassium 20mg/10mmol m/r tablet; inspra 25mg tablet; spiroprop tablet; metoros 95mg tablet; bedranol 10mg tablet; totamol 25mg tablet; arbralene 50mg tablet; kerlone 20mg tablet; trandate 50mg tablet; betaloc 50mg tablet; mepranix 50mg tablet; metoprolol; artrate+chlorthalidone 100mg/12.5mg tablet; secadrex tablet; sotazide tablet; penbutolol sulphate+frusemide 40mg/20mg tablet; tenoret 50 tablet; tenoretic tablet; tolerzide tablet; viskaldix tablet; timolol maleate+bendrofluazide 10mg/2.5mg tablet; timolol maleate+bendrofluazide 20mg/5mg tablet; atenixco 50mg/12.5mg tablet; beta-adalat capsule; tenif capsule; co-betaloc tablet; inderetic capsule; inderex capsule; kalten capsule; lopresoretic tablet; moducren tablet; prestim tablet; atenolol+nifedipine 50mg/20mg m/r capsule; monocor 5mg tablet; emcor 10mg tablet; celectol 200mg tablet; adalat 5mg capsule; monozide 10 tablet; antipressan 25mg tablet; angilol 10mg tablet; cardinol 10mg tablet; acebutolol; atenolol; tenormin 25 tablet; vasaten 50mg tablet; apsolol 10mg tablet; propanix 10mg tablet; betadur cr 160mg m/r capsule; beta-prograne 160mg m/r capsule; berkolol 10mg tablet; half-betadur cr 80mg m/r capsule; half-inderal la 80mg m/r capsule; half beta-prograne 80mg m/r capsule; inderal 10mg tablet; concordin 5mg tablet; betaxolol; bisoprolol; celiprolol; metoprolol; labetalol; penbutolol; sotalol; timolol; adalate 10mg capsule; avlocardyl retard 160mg m/r capsule; carvedilol; totaretic 50mg/12.5mg tablet; sloprolol 80mg m/r capsule; probeta la 160mg m/r capsule; lopranol la 160mg m/r capsule; atenix 25mg tablet; co-tenidone; atenolol+chlorthalidone; atenolol+bendrofluazide; tenben capsule; half propanix la 80mg m/r capsule; half propatard la 80mg m/r capsule; nebilet 5mg tablet; eucardic 3.125 tablet; dorzolamide+timolol; cardicor 1.25mg tablet; syprol 5mg/5ml oral solution; soloc 5mg tablet; tensomex 100mg tablet; bipranix 5mg tablet; latanoprost+timolol; rapranol sr 80mg m/r capsule; vivacor 5mg tablet;calcicard 60mg tablet; lidoflazine; clinium 120mg tablet; vasad 5mg capsule; synadrin 60mg tablet; veractil 25mg tablet; nifedipine; calcilat 10mg capsule; angiopine 5mg capsule; nifensar xl 20mg m/r tablet; coracten sr 10mg m/r capsule; tildiem 60mg m/r tablet; britiazim 60mg m/r tablet; angiozem 60mg m/r tablet; adizem-60 m/r tablet; cardene 20mg capsule; isradipine; istin 5mg tablet; lacidipine; motens 2mg tablet; half securon sr 120mg m/r tablet; securon 40mg tablet; geangin 40mg tablet; berkatens 40mg tablet; cordilox 40mg tablet; parmid 10mg tablet; nimotop 30mg tablet; nimodipine; nicardipine; univer 120mg m/r capsule; verapamil; nifelease 20mg m/r tablet; slozem 120mg m/r capsule; calanif 5mg capsule; angitil sr 90 m/r capsule; adipine mr 10 m/r tablet; kentiazem 60mg m/r capsule; unipine xl 30mg m/r tablet; adizem-xl plus m/r capsule; cardilate mr 10mg m/r tablet; tensipine mr 10 m/r tablet; plendil 2.5mg m/r tablet; nisoldipine; syscor mr 10mg m/r tablet; fortipine la40 m/r tablet; slofedipine 20mg m/r tablet; nifedotard 20mr m/r tablet; verapress mr 240 m/r tablet; viazem xl 120mg m/r capsule; genalat retard 10mg m/r tablet; lercanidipine; zanidip 10mg tablet; mibefradil; calazem 60mg m/r tablet; optil 60mg m/r tablet; dilcardia sr 60mg m/r capsule; nifedipress mr 10 m/r tablet; nivaten retard 10mg m/r tablet; coroday mr 20mg m/r tablet; zemtard 120 xl m/r capsule; ethimil mr 240 m/r tablet; vertab sr 240 m/r tablet; nifopress retard 20mg m/r tablet; zildil sr 60mg m/r capsule; calchan mr 10mg m/r tablet; zemret 180 xl m/r capsule; bi-carzem sr 60mg m/r capsule; horizem sr 90mg m/r capsule; zolvera 40mg/5ml oral solution; disogram sr 60mg m/r capsule; ranvera mr 240mg m/r tablet; cabren 2.5mg m/r tablet; vera-til sr 120mg m/r tablet; kentipine mr 10mg m/r tablet; felotens xl 5mg m/r tablet; felogen xl 5mg m/r tablet; hypolar retard 10mg m/r tablet; valni 20 retard 20mg m/r tablet; cardioplen xl 5mg m/r tablet; amlostin 5mg tablet; neofel xl 5mg m/r tablet; parmid xl 5mg m/r tablet.  1140851692; 1140860696; 1140860706; 1140860714; 1140860728; 1140860736; 1140860750; 1140860752; 1140860758; 1140860764; 1140860776; 1140860784; 1140860790; 1140860802; 1140860806; 1140860878; 1140860882; 1140860892; 1140860904; 1140860912; 1140860918; 1140864618; 1140864910; 1140864952; 1140866078; 1140879802; 1140881706; 1140888552; 1140888556; 1140888560; 1140888646; 1140916356; 1140916362; 1140923718; 1141145660; 1141145668; 1141150328; 1141150560; 1141151016; 1141151018; 1141152998; 1141153006; 1141153316; 1141153328; 1141156836; 1141156846; 1141164148; 1141164154; 1141165470; 1141165476; 1141166006; 1141167758; 1141167822; 1141170544; 1141170870; 1141171336; 1141172492; 1141172682; 1141172686; 1141179974; 1141180592; 1141180598; 1141187788; 1141188408; 1141190934; 1141193282; 1141193346; 1141199940; 1141200698; 1141200726; 1141201038; 1141201040; 1140851332; 1140851336; 1140851338; 1140851362; 1140851364; 1140851368; 1140851660; 1140860332; 1140860404; 1140860422; 1140860562; 1140860738; 1140864950; 1140866072; 1140866074; 140866084; 1140866086; 1140866092; 1140866096; 1140866108; 1140866110; 1140866128; 1140866132; 1140866136; 1140866138; 1140866140; 1140866146; 1140866156; 1140866158; 1140866162; 1140866164; 1140866168; 1140866422; 1140866440; 1140866446; 1140888918; 1140888922; 1140909706; 1140916870; 1140917068; 1140926778; 1141146378; 1141180772; 1141180778; 1141188636; 1141194794; 1141194800; 1141194804; 1141194808; 1141194810; 1140851360; 1140851418; 1140851420; 1140851428; 1140851430; 1140851436; 1140860292; 1140860322; 1140860336;1140864550; 1140864574; 1140866220; 1140866226; 1140866232; 1140866236; 1140866244; 1140866248; 1140866280; 1140866282; 1140866306; 1140866308; 1140866312; 1140866318; 1140866324; 1140866328; 1140866330; 1140866332; 1140866334; 1140866340; 1140866352; 1140866354; 1140866356; 1140866360; 1140866388; 1140866390; 1140866396; 1140866400; 1140866402; 1140866404; 1140866406; 1140866410; 1140866416; 1140866418; 1140866420; 1140866426; 1140866438; 1140866442; 1140866444; 1140866448; 1140881894; 1140888512; 1140909708; 1140909722; 1140922324; 1140923272; 1140923276; 1140923282; 1140923402; 1140927174; 1140928624; 1141146128; 1141150898; 1141167108; 1141181520; 1141195254; 1141195258; 1141201250; 1140851508; 1140851522; 1140851556; 1140860172; 1140860180; 1140860232; 1140860250; 1140860266; 1140860278; 1140860308; 1140860314; 1140860318; 1140860320; 1140860324; 1140860328; 1140860330; 1140860338; 1140860340; 1140860342; 1140860348; 1140860356; 1140860358; 1140860386; 1140860394; 1140860396; 1140860398; 1140860402; 1140860406; 1140860410; 1140860426; 1140860434; 1140860492; 1140860498; 1140861090; 1140864176; 1140864410; 1140866704; 1140866712; 1140866724; 1140866738; 1140866756; 1140866758; 1140866764; 1140866766; 1140866778; 1140866782; 1140866784; 1140866798; 1140866800; 1140866802; 1140866804; 1140867734; 1140879758; 1140879760; 1140879762; 1140879818; 1140879824; 1140879834; 1140879854; 1140879866; 1140881702; 1140881722; 1140909368; 1140916628; 1140916730; 1140916868; 1140917076; 1140922930; 1140923336; 1141146124; 1141146126; 1141146184; 1141152076; 1141156754; 1141164280; 1141168498; 1141169516; 1141171152; 1141172742; 1141182904; 1141182968; 1141184324; 1141184722; 1141187048; 1141187780; 1140851730; 1140851784; 1140851786; 1140851790; 1140851800; 1140855976; 1140861088; 1140861106; 1140861110; 1140861114; 1140861120; 1140861128; 1140861130; 1140861136; 1140861138; 1140861176; 1140861190; 1140861202; 1140861276; 1140861282; 1140866460; 1140866466; 1140866484; 1140866546; 1140866554; 1140868036; 1140872472; 1140872568; 1140879810; 1140881692; 1140888510; 1140911088; 1140911698; 1140916930; 1140917428; 1140923572; 1140923618; 1140926188; 1140926780; 1140927934; 1140927940; 1140928212; 1140928226; 1140928234; 1141145870; 1141150500; 1141150538; 1141150926; 1141151474; 1141152600; 1141153026; 1141153032; 1141153394; 1141153454; 1141156656; 1141157136; 1141157140; 1141162546; 1141166752; 1141167832; 1141169096; 1141169710; 1141169730; 1141171804; 1141173766; 1141174684; 1141175224; 1141180238; 1141184390; 1141185444; 1141187056; 1141187094; 1141187774; 1141187962; 1141188152; 1141188576; 1141188936; 1141190548; 1141199858; 1141200400; 1141200782; 1141201814 | |
| Antidiabetic medication | 20003; 6177; 6153 |
| Glibornuride; daonil 5mg tablet; glutril 25mg tablet; semi-daonil 2.5mg tablet; glymidine; euglucon 2.5mg tablet; gondafon 500mg tablet ; malix 2.5mg tablet ; pramidex 500mg tablet; diabetamide 2.5mg tablet; glipizide; gliclazide; glibenese 5mg tablet; diamicron 80mg tablet; minodiab 2.5mg tablet; insulin product; tolazamide; metformin; tolanase 100mg tablet; glimepiride; tolbutamide; troglitazone; glyconon 500mg tablet; romozin 200mg tablet ; rastinon 500mg tablet ; amaryl 1mg tablet ; glucophage 500mg tablet; pioglitazone; orabet 500mg tablet; actos 15mg tablet; chlorpropamide; rosiglitazone; diabinese 100mg tablet; avandia 4mg tablet; glymese 250mg tablet; rosiglitazone 1mg / metformin 500mg tablet; glibenclamide; avandamet 1mg / 500mg tablet  1140857494; 1140874724; 1140857496; 1140874726;1140857500;1140874728; 1140857502; 1140874732; 1140857506; 1140874736; 1140874646; 1140874744; 1140874650; 1140874746; 1140874652; 1140883066; 1140874664; 1140884600; 1140874666; 1141152590; 1140874674; 1141153254; 1140874678; 1141153262; 1140874680; 1141156984; 1140874686; 1141171646; 1140874690; 1141171652; 1140874706; 1141177600; 1140874712; 1141177606; 1140874716; 1141189090; 1140874718; 1141189094; | |

**Supplementary Table S3. Baseline characteristics involved in the research**

| Variable | Level | Overall1 |
| --- | --- | --- |
| Sample size, No. | - | 18693 |
| Age (mean (SD)) | - | 57.28 (7.78) |
| Race (%) | Others | 810 (4.3) |
|  | White | 17883 (95.7) |
| Sex (%) | Female | 12540 (67.1) |
|  | Male | 6153 (32.9) |
| Townsend index (mean (SD)) | - | -1.29 (3.11) |
| BMI (mean (SD)) | - | 26.92 (4.61) |
| MET-minutes/week (mean [SD]) | - | 2619.88 (2634.68) |
| Sleep duration (mean (SD)) | - | 7.10 (1.39) |
| Smoke (%) | Current | 2314 (12.4) |
|  | Never | 9518 (50.9) |
|  | Previous | 6771 (36.2) |
|  | Unknown | 90 (0.5) |
| Alcohol (%) | Current | 16996 (90.9) |
|  | Never | 857 (4.6) |
|  | Previous | 795 (4.3) |
|  | Unknown | 45 (0.2) |
| Hypertension (%) | No | 18441 (98.7) |
|  | Yes | 252 (1.3) |
| Glucose concentration (mean (SD)) | - | 5.01 (0.85) |
| HbA1c concentration (mean (SD)) | - | 35.43 (4.87) |
| Creatinine (mean (SD)) | - | 69.70 (16.59) |
| CRP aliquot (mean (SD)) | - | 1.10 (0.43) |
| Cholesterol (mean (SD)) | - | 5.86 (1.12) |
| HDL Cholesterol (mean (SD)) | - | 1.51 (0.40) |
| Triglycerides (mean (SD)) | - | 1.70 (0.99) |
| Follow up examination after treatment (%) | No | 18417 (98.5) |
|  | Yes | 276 (1.5) |
| Chemotherapy session for neoplasm (%) | No | 9292 (49.7) |
|  | Yes | 9401 (50.3) |
| Other chemotherapy (%) | No | 11485 (61.4) |
|  | Yes | 7208 (38.6) |
| Personal history of chemotherapy (%) | No | 12990 (69.5) |
|  | Yes | 5703 (30.5) |
| Convalescence following chemotherapy (%) | No | 18690 (100.0) |
|  | Yes | 3 (0.0) |
| Cancer (%) | Bladder | 439 (2.3) |
|  | Brain | 203 (1.1) |
|  | Breast | 6638 (35.5) |
|  | Colorectal | 2812 (15.0) |
|  | Endometrium | 384 (2.1) |
|  | Esophagus | 500 (2.7) |
|  | Hodgkin | 225 (1.2) |
|  | Kidney | 159 (0.9) |
|  | Leukemia | 657 (3.5) |
|  | Liver | 86 (0.5) |
|  | Lung | 1105 (5.9) |
|  | Malignant melanoma | 383 (2.0) |
|  | Multiple myeloma | 537 (2.9) |
|  | Non-Hodgkin | 1484 (7.9) |
|  | Oral | 568 (3.0) |
|  | Ovarian | 794 (4.2) |
|  | Pancreas | 331 (1.8) |
|  | Prostate | 993 (5.3) |
|  | Stomach | 309 (1.7) |
|  | Thyroid | 86 (0.5) |
| Delivery of a fraction of external beam radiotherapy NEC (%) | No | 17952 (96.0) |
|  | Yes | 741 (4.0) |
| Megavoltage treatment for adaptive radiotherapy (%) | No | 18593 (99.5) |
|  | Yes | 100 (0.5) |
| Oral delivery of radiotherapy for thyroid ablation (%) | No | 18670 (99.9) |
|  | Yes | 23 (0.1) |
| Preparation for simple radiotherapy with imaging and dosimetry(%) | No | 18657 (99.8) |
|  | Yes | 36 (0.2) |
| Megavoltage treatment for simple radiotherapy (%) | No | 18454 (98.7) |
|  | Yes | 239 (1.3) |
| Preparation for complex conformal radiotherapy(%) | No | 18561 (99.3) |
|  | Yes | 132 (0.7) |
| Technical support for preparation for radiotherapyt (%) | No | 18576 (99.4) |
|  | Yes | 117 (0.6) |
| Unspecified external beam radiotherapy (%) | No | 18569 (99.3) |
|  | Yes | 124 (0.7) |
| Unspecified preparation for external beam radiotherap (%) | No | 18647 (99.8) |
|  | Yes | 46 (0.2) |
| Delivery of a fraction of interstitial radiotherapy (%) | No | 18645 (99.7) |
|  | Yes | 48 (0.3) |
| Preparation for simple radiotherapy with imaging and simple calculation (%) | No | 18529 (99.1) |
|  | Yes | 164 (0.9) |
| Unspecified support for preparation for radiotherapy (%) | No | 18678 (99.9) |
|  | Yes | 15 (0.1) |
| Preparation for superficial radiotherapy with simple calculation (%) | No | 18687 (100.0) |
|  | Yes | 6 (0.0) |
| Unspecified radiotherapy delivery (%) | No | 18593 (99.5) |
|  | Yes | 100 (0.5) |
| Delivery of a fraction of intracavitary radiotherapy (%) | No | 18610 (99.6) |
|  | Yes | 83 (0.4) |
| Other specified radiotherapy delivery (%) | No | 18643 (99.7) |
|  | Yes | 50 (0.3) |
| Superficial or orthovoltage treatment for radiotherapy (%) | No | 18688 (100.0) |
|  | Yes | 5 (0.0) |
| Megavoltage treatment for hypofractionated stereotactic radiotherapy (%) | No | 18685 (100.0) |
|  | Yes | 8 (0.0) |
| Other specified support for preparation for radiotherapy (%) | No | 18690 (100.0) |
|  | Yes | 3 (0.0) |
| Lipid-lowering medication (%) | No | 17048 (91.2) |
|  | Yes | 1645 (8.8) |
| Anti-hypertensive medication (%) | No | 17205 (92.0) |
|  | Yes | 1488 (8.0) |
| Anti-diabetic medication (%) | No | 18526 (99.1) |
|  | Yes | 167 (0.9) |

Abbreviation: BMI, Body mass index; CRP, C-Reactive Protein; HDL, High Density Protein; HbA1c, haemoglobin A1c; MET, metabolic equivalent of task.

^1^ Continuous variables presented as mean (standard deviation); categorical variables as n (%).

**Supplementary Table S4. Cluster variables based on UMAP-HDBSAN.**

| Variable | Cluster1 | Cluster2 | Cluster3 | Cluster4 | Cluster5 | Cluster6 | Cluster7 | Cluster8 | Cluster9 |
| --- | --- | --- | --- | --- | --- | --- | --- | --- | --- |
| CRP aliquot | 1.12 | 1.08 | 1.11 | 1.12 | 1.12 | 1.07 | 1.13 | 1.09 | 1.07 |
| Cancer | Bladder: 0.8%, Brain: 0.9%, Breast: 48%, Colorectal: 12%, Endometrium: 3%, Esophagus: 1.7%, Hodgkin: 0.8%, Kidney: 1%, Leukemia: 2.1%, Liver: 0.5%, Lung: 6.7%, Malignant melanoma: 2.9%, Multiple myeloma: 2.6%, Non-Hodgkin: 6.2%, Oral: 1.7%, Ovarian: 6%, Pancreas: 1.4%, Prostate: 0%, Stomach: 0.8%, Thyroid: 0.9% | Bladder: 5.4%, Brain: 1.7%, Breast: 0.4%, Colorectal: 20%, Endometrium: 0%, Esophagus: 5%, Hodgkin: 1.3%, Kidney: 1.1%, Leukemia: 7.1%, Liver: 0.4%, Lung: 7%, Malignant melanoma: 2.9%, Multiple myeloma: 3.9%, Non-Hodgkin: 10.4%, Oral: 6.1%, Ovarian: 0%, Pancreas: 2.7%, Prostate: 21.8%, Stomach: 2.7%, Thyroid: 0.2% | Bladder: 0.9%, Brain: 1.1%, Breast: 91.7%, Colorectal: 0%, Endometrium: 0%, Esophagus: 0.9%, Hodgkin: 0%, Kidney: 0.9%, Leukemia: 0%, Liver: 0.3%, Lung: 0.1%, Malignant melanoma: 0%, Multiple myeloma: 1.7%, Non-Hodgkin: 0%, Oral: 0.7%, Ovarian: 0%, Pancreas: 0%, Prostate: 0%, Stomach: 0.9%, Thyroid: 0.7% | Bladder: 1.1%, Brain: 0.5%, Breast: 67%, Colorectal: 3.2%, Endometrium: 3.7%, Esophagus: 2%, Hodgkin: 1.2%, Kidney: 0.4%, Leukemia: 3.4%, Liver: 0.7%, Lung: 7.6%, Malignant melanoma: 1%, Multiple myeloma: 1.1%, Non-Hodgkin: 0.7%, Oral: 1.6%, Ovarian: 1.7%, Pancreas: 1.5%, Prostate: 0%, Stomach: 1.1%, Thyroid: 0.4% | Bladder: 0.9%, Brain: 0.6%, Breast: 56.3%, Colorectal: 10.6%, Endometrium: 2.7%, Esophagus: 1%, Hodgkin: 0.8%, Kidney: 0.4%, Leukemia: 1.9%, Liver: 0.1%, Lung: 4%, Malignant melanoma: 1%, Multiple myeloma: 2.1%, Non-Hodgkin: 7.7%, Oral: 2.1%, Ovarian: 6.2%, Pancreas: 0.6%, Prostate: 0%, Stomach: 0.5%, Thyroid: 0.2% | Bladder: 5.1%, Brain: 1.4%, Breast: 0.6%, Colorectal: 25.7%, Endometrium: 0%, Esophagus: 5.1%, Hodgkin: 2.5%, Kidney: 1.2%, Leukemia: 6.3%, Liver: 0.5%, Lung: 7.4%, Malignant melanoma: 1.9%, Multiple myeloma: 4.7%, Non-Hodgkin: 13.8%, Oral: 6.3%, Ovarian: 0%, Pancreas: 2.8%, Prostate: 10.4%, Stomach: 4%, Thyroid: 0.3% | Bladder: 1.2%, Brain: 1%, Breast: 46.7%, Colorectal: 14.2%, Endometrium: 3.1%, Esophagus: 1.4%, Hodgkin: 0.2%, Kidney: 0.5%, Leukemia: 2.1%, Liver: 0.7%, Lung: 6.8%, Malignant melanoma: 2.1%, Multiple myeloma: 3.1%, Non-Hodgkin: 4.1%, Oral: 2.7%, Ovarian: 5.8%, Pancreas: 2.6%, Prostate: 0%, Stomach: 1.4%, Thyroid: 0.3% | Bladder: 0.6%, Brain: 1.5%, Breast: 49.5%, Colorectal: 12.6%, Endometrium: 4.4%, Esophagus: 1.7%, Hodgkin: 0.8%, Kidney: 0.6%, Leukemia: 2.9%, Liver: 0.7%, Lung: 3.1%, Malignant melanoma: 2.9%, Multiple myeloma: 3%, Non-Hodgkin: 6.1%, Oral: 1.1%, Ovarian: 5.5%, Pancreas: 1.9%, Prostate: 0%, Stomach: 0.8%, Thyroid: 0.6% | Bladder: 6.2%, Brain: 2.4%, Breast: 0.1%, Colorectal: 22.5%, Endometrium: 0%, Esophagus: 6.2%, Hodgkin: 0.7%, Kidney: 1.7%, Leukemia: 4.7%, Liver: 1.2%, Lung: 5.8%, Malignant melanoma: 2.1%, Multiple myeloma: 3.9%, Non-Hodgkin: 9.7%, Oral: 5.8%, Ovarian: 0%, Pancreas: 3%, Prostate: 20.8%, Stomach: 3%, Thyroid: 0.1% |
| Chemotherapy session for neoplasm | No: 97.3%, Yes: 2.7% | No: 99.4%, Yes: 0.6% | No: 0%, Yes: 100% | No: 0%, Yes: 100% | No: 0%, Yes: 100% | No: 0.1%, Yes: 99.9% | No: 100%, Yes: 0% | No: 100%, Yes: 0% | No: 100%, Yes: 0% |
| Cholesterol | 5.94 | 5.6 | 6 | 6 | 5.99 | 5.62 | 6.11 | 5.95 | 5.73 |
| Creatinine | 63.68 | 81.07 | 63.83 | 63.96 | 64.34 | 81.57 | 64.38 | 64.38 | 80.2 |
| Follow up examination after treatment | No: 99.9%, Yes: 0.1% | No: 99.8%, Yes: 0.2% | No: 99.7%, Yes: 0.3% | No: 98.7%, Yes: 1.3% | No: 98.5%, Yes: 1.5% | No: 97.1%, Yes: 2.9% | No: 99.1%, Yes: 0.9% | No: 98.6%, Yes: 1.4% | No: 97.9%, Yes: 2.1% |
| Glucose concentration | 4.97 | 5 | 5.02 | 5.04 | 5.02 | 5.08 | 4.93 | 4.94 | 4.97 |
| HDL Cholesterol | 1.61 | 1.28 | 1.65 | 1.63 | 1.62 | 1.3 | 1.67 | 1.63 | 1.33 |
| HbA1c concentration | 35.25 | 35.31 | 35.12 | 35.67 | 35.36 | 35.9 | 34.82 | 34.62 | 34.76 |
| Other chemotherapy | No: 0%, Yes: 100% | No: 0%, Yes: 100% | No: 100%, Yes: 0% | No: 100%, Yes: 0% | No: 87.9%, Yes: 12.1% | No: 90.2%, Yes: 9.8% | No: 100%, Yes: 0% | No: 100%, Yes: 0% | No: 100%, Yes: 0% |
| Personal history of chemotherapy | No: 97.1%, Yes: 2.9% | No: 96.6%, Yes: 3.4% | No: 100%, Yes: 0% | No: 100%, Yes: 0% | No: 0.5%, Yes: 99.5% | No: 72.5%, Yes: 27.5% | No: 0%, Yes: 100% | No: 0%, Yes: 100% | No: 0%, Yes: 100% |
| Age | 56.70 | 56.36 | 55.69 | 57.13 | 56.88 | 59.39 | 57.69 | 55.46 | 57.58 |
| Alcohol | Current: 87.9%, Never: 6.2%, Previous: 5.7%, Unknown: 0.3% | Current: 93.1%, Never: 2.8%, Previous: 3.8%, Unknown: 0.3% | Current: 100%, Never: 0%, Previous: 0%, Unknown: 0% | Current: 89.6%, Never: 5.4%, Previous: 4.4%, Unknown: 0.6% | Current: 90.8%, Never: 5%, Previous: 4%, Unknown: 0.2% | Current: 93%, Never: 2.1%, Previous: 4.7%, Unknown: 0.3% | Current: 94.7%, Never: 2.2%, Previous: 3.1%, Unknown: 0% | Current: 91.1%, Never: 6.4%, Previous: 2%, Unknown: 0.4% | Current: 94.5%, Never: 2.5%, Previous: 2.9%, Unknown: 0% |
| Hypertension | No: 99.5%, Yes: 0.5% | No: 99.4%, Yes: 0.6% | No: 99.7%, Yes: 0.3% | No: 99.7%, Yes: 0.3% | No: 98.4%, Yes: 1.6% | No: 96.9%, Yes: 3.1% | No: 100%, Yes: 0% | No: 100%, Yes: 0% | No: 100%, Yes: 0% |
| BMI | 26.97 | 27.72 | 26.18 | 26.81 | 26.67 | 27.32 | 26.4 | 25.82 | 26.75 |
| Anti-diabetic medication | No: 99.4%, Yes: 0.6% | No: 98.7%, Yes: 1.3% | No: 99.4%, Yes: 0.6% | No: 99.5%, Yes: 0.5% | No: 99.6%, Yes: 0.4% | No: 98.5%, Yes: 1.5% | No: 100%, Yes: 0% | No: 100%, Yes: 0% | No: 100%, Yes: 0% |
| Anti-hypertensive medication | No: 92.2%, Yes: 7.8% | No: 92.3%, Yes: 7.7% | No: 100%, Yes: 0% | No: 99.7%, Yes: 0.3% | No: 93.1%, Yes: 6.9% | No: 87.9%, Yes: 12.1% | No: 99.3%, Yes: 0.7% | No: 99.3%, Yes: 0.7% | No: 98.8%, Yes: 1.2% |
| Lipid-lowering medication | No: 92.2%, Yes: 7.8% | No: 87.1%, Yes: 12.9% | No: 100%, Yes: 0% | No: 92.1%, Yes: 7.9% | No: 94%, Yes: 6% | No: 87.5%, Yes: 12.5% | No: 94.9%, Yes: 5.1% | No: 97.3%, Yes: 2.7% | No: 92.8%, Yes: 7.2% |
| Race | others: 5.1%, white: 94.9% | others: 5.2%, white: 94.8% | others: 0%, white: 100% | others: 8.9%, white: 91.1% | others: 4.6%, white: 95.4% | others: 3.4%, white: 96.6% | others: 3.1%, white: 96.9% | others: 5.7%, white: 94.3% | others: 3.3%, white: 96.7% |
| Delivery of a fraction of external beam radiotherapy NEC | No: 99.3%, Yes: 0.7% | No: 99%, Yes: 1% | No: 100%, Yes: 0% | No: 96.9%, Yes: 3.1% | No: 93.6%, Yes: 6.4% | No: 95.2%, Yes: 4.8% | No: 97.4%, Yes: 2.6% | No: 95.8%, Yes: 4.2% | No: 94.3%, Yes: 5.7% |
| Delivery of a fraction of interstitial radiotherapy | No: 100%, Yes: 0% | No: 99.6%, Yes: 0.4% | No: 100%, Yes: 0% | No: 99.9%, Yes: 0.1% | No: 99.7%, Yes: 0.3% | No: 99.6%, Yes: 0.4% | No: 99.5%, Yes: 0.5% | No: 99.7%, Yes: 0.3% | No: 98.9%, Yes: 1.1% |
| Delivery of a fraction of intracavitary radiotherapy | No: 99.9%, Yes: 0.1% | No: 99.9%, Yes: 0.1% | No: 99.9%, Yes: 0.1% | No: 99%, Yes: 1% | No: 98.7%, Yes: 1.3% | No: 100%, Yes: 0% | No: 98.8%, Yes: 1.2% | No: 98.9%, Yes: 1.1% | No: 99.7%, Yes: 0.3% |
| Megavoltage treatment for simple radiotherapy | No: 99.9%, Yes: 0.1% | No: 99.9%, Yes: 0.1% | No: 100%, Yes: 0% | No: 99.7%, Yes: 0.3% | No: 99.4%, Yes: 0.6% | No: 99.4%, Yes: 0.6% | No: 99.7%, Yes: 0.3% | No: 99.9%, Yes: 0.1% | No: 99.7%, Yes: 0.3% |
| Oral delivery of radiotherapy for thyroid ablation | No: 99.9%, Yes: 0.1% | No: 99.8%, Yes: 0.2% | No: 99.7%, Yes: 0.3% | No: 99.8%, Yes: 0.2% | No: 99.7%, Yes: 0.3% | No: 99.9%, Yes: 0.1% | No: 100%, Yes: 0% | No: 99.9%, Yes: 0.1% | No: 100%, Yes: 0% |
| Other specified radiotherapy delivery | No: 99.9%, Yes: 0.1% | No: 100%, Yes: 0% | No: 99.6%, Yes: 0.4% | No: 99.1%, Yes: 0.9% | No: 99.8%, Yes: 0.2% | No: 99.4%, Yes: 0.6% | No: 99.8%, Yes: 0.2% | No: 100%, Yes: 0% | No: 99.9%, Yes: 0.1% |
| Preparation for complex conformal radiotherapy | No: 99.8%, Yes: 0.2% | No: 99.9%, Yes: 0.1% | No: 100%, Yes: 0% | No: 99.8%, Yes: 0.2% | No: 98.2%, Yes: 1.8% | No: 99.4%, Yes: 0.6% | No: 100%, Yes: 0% | No: 98.3%, Yes: 1.7% | No: 98.5%, Yes: 1.5% |
| Preparation for simple radiotherapy with imaging and simple calculation | No: 99.9%, Yes: 0.1% | No: 99.9%, Yes: 0.1% | No: 100%, Yes: 0% | No: 100%, Yes: 0% | No: 99.7%, Yes: 0.3% | No: 99.9%, Yes: 0.1% | No: 99.7%, Yes: 0.3% | No: 100%, Yes: 0% | No: 99.7%, Yes: 0.3% |
| Preparation for simple radiotherapy with imaging and dosimetry | No: 100%, Yes: 0% | No: 100%, Yes: 0% | No: 100%, Yes: 0% | No: 100%, Yes: 0% | No: 99.9%, Yes: 0.1% | No: 99.8%, Yes: 0.2% | No: 100%, Yes: 0% | No: 100%, Yes: 0% | No: 99.7%, Yes: 0.3% |
| Technical support for preparation for radiotherapy | No: 99.8%, Yes: 0.2% | No: 99.8%, Yes: 0.2% | No: 100%, Yes: 0% | No: 99.8%, Yes: 0.2% | No: 98.5%, Yes: 1.5% | No: 99.5%, Yes: 0.5% | No: 99.3%, Yes: 0.7% | No: 99.6%, Yes: 0.4% | No: 98.2%, Yes: 1.8% |
| Unspecified radiotherapy delivery | No: 99.9%, Yes: 0.1% | No: 100%, Yes: 0% | No: 99.3%, Yes: 0.7% | No: 98.5%, Yes: 1.5% | No: 99.8%, Yes: 0.2% | No: 98.5%, Yes: 1.5% | No: 99.7%, Yes: 0.3% | No: 100%, Yes: 0% | No: 100%, Yes: 0% |
| Unspecified external beam radiotherapy | No: 99.9%, Yes: 0.1% | No: 99.9%, Yes: 0.1% | No: 100%, Yes: 0% | No: 99.3%, Yes: 0.7% | No: 98.2%, Yes: 1.8% | No: 98.5%, Yes: 1.5% | No: 100%, Yes: 0% | No: 99.6%, Yes: 0.4% | No: 99.3%, Yes: 0.7% |
| Unspecified preparation for external beam radiotherapy | No: 100%, Yes: 0% | No: 100%, Yes: 0% | No: 100%, Yes: 0% | No: 99.8%, Yes: 0.2% | No: 99.5%, Yes: 0.5% | No: 99.5%, Yes: 0.5% | No: 100%, Yes: 0% | No: 99.9%, Yes: 0.1% | No: 99.8%, Yes: 0.2% |
| Sex | Female: 100%, Male: 0% | Female: 0%, Male: 100% | Female: 100%, Male: 0% | Female: 99.9%, Male: 0.1% | Female: 100%, Male: 0% | Female: 0%, Male: 100% | Female: 100%, Male: 0% | Female: 99.5%, Male: 0.5% | Female: 0%, Male: 100% |
| Sleep duration | 7.00 | 6.99 | 7.24 | 7.10 | 7.12 | 7.21 | 7.19 | 7.15 | 7.08 |
| Smoke | Current: 12.3%, Never: 53.5%, Previous: 33.5%, Unknown: 0.7% | Current: 15.6%, Never: 42.8%, Previous: 41.1%, Unknown: 0.5% | Current: 0%, Never: 99.6%, Previous: 0%, Unknown: 0.4% | Current: 0.4%, Never: 16.7%, Previous: 82.3%, Unknown: 0.6% | Current: 10.1%, Never: 53.9%, Previous: 35.6%, Unknown: 0.4% | Current: 15.5%, Never: 40.4%, Previous: 43.6%, Unknown: 0.5% | Current: 0.2%, Never: 0%, Previous: 99.7%, Unknown: 0.2% | Current: 0.3%, Never: 98.9%, Previous: 0.1%, Unknown: 0.7% | Current: 16.5%, Never: 47.3%, Previous: 36.3%, Unknown: 0% |
| Townsend index | -1.25 | -1.17 | -1.75 | -1.02 | -1.26 | -1.25 | -1.46 | -1.83 | -1.42 |

Abbreviation: UMAP:Uniform Manifold Approximation and Project;HDBSCAN, Hierarchical Density-Based Spatial Clustering of Applications with Noise ;CRP,C-Reactive Protein; HDL,High-density lipoprotein ;Hb A1c,haemoglobin A1c;BMI,Body mass index

**Supplementary Table S5. Baseline characteristics of noise group compared to the core clustered population.**

|  | **Overall** | **Core Group (Clusters 1-9)** | **Noise Group (Cluster 0)** | ***P*** | **SMD** |
| --- | --- | --- | --- | --- | --- |
| N | 18693 | 16107 | 2586 |  |  |
| Age (mean (SD)) | 57.28 (7.78) | 57.17 (7.82) | 57.95 (7.48) | <0.001 | 0.102 |
| Race = white (%) | 17883 (95.7) | 15379 (95.5) | 2504 (96.8) | 0.002 | 0.07 |
| Sex = Male (%) | 6153 (32.9) | 6036 (37.5) | 117 (4.5) | <0.001 | 0.885 |
| Townsend index (mean (SD)) | -1.29 (3.11) | -1.31 (3.10) | -1.13 (3.14) | 0.007 | 0.057 |
| BMI (mean (SD)) | 26.92 (4.61) | 26.92 (4.58) | 26.93 (4.80) | 0.93 | 0.002 |
| MET (mean (SD)) | 2619.88 (2634.68) | 2637.08 (2654.23) | 2512.77 (2507.27) | 0.026 | 0.048 |
| Sleep duration (mean (SD)) | 7.10 (1.39) | 7.10 (1.38) | 7.14 (1.45) | 0.152 | 0.03 |
| Smoke (%) |  |  |  | <0.001 | 0.439 |
| Current | 2314 (12.4) | 1683 (10.4) | 631 (24.4) |  |  |
| Never | 9518 (50.9) | 8178 (50.8) | 1340 (51.8) |  |  |
| Previous | 6771 (36.2) | 6165 (38.3) | 606 (23.4) |  |  |
| unknown | 90 (0.5) | 81 (0.5) | 9 (0.3) |  |  |
| Alcohol (%) |  |  |  | <0.001 | 0.174 |
| Current | 16996 (90.9) | 14751 (91.6) | 2245 (86.8) |  |  |
| Never | 857 (4.6) | 653 (4.1) | 204 (7.9) |  |  |
| Previous | 795 (4.3) | 662 (4.1) | 133 (5.1) |  |  |
| Unknown | 45 (0.2) | 41 (0.3) | 4 (0.2) |  |  |
| Hypertension = yes (%) | 252 (1.3) | 163 (1.0) | 89 (3.4) | <0.001 | 0.165 |
| Glucose concentration (mean (SD)) | 5.01 (0.85) | 5.01 (0.84) | 5.06 (0.92) | 0.003 | 0.061 |
| HbA1c concentration (mean (SD)) | 35.43 (4.87) | 35.33 (4.85) | 36.05 (4.90) | <0.001 | 0.147 |
| Creatinine (mean (SD)) | 69.70 (16.59) | 70.40 (16.56) | 65.30 (16.08) | <0.001 | 0.312 |
| CRP aliquot (mean (SD)) | 1.10 (0.43) | 1.10 (0.43) | 1.11 (0.46) | 0.595 | 0.011 |
| Cholesterol (mean (SD)) | 5.86 (1.12) | 5.85 (1.11) | 5.96 (1.16) | <0.001 | 0.104 |
| HDL Cholesterol (mean (SD)) | 1.51 (0.40) | 1.50 (0.40) | 1.55 (0.38) | <0.001 | 0.13 |
| Triglycerides (mean (SD)) | 1.70 (0.99) | 1.70 (1.00) | 1.69 (0.93) | 0.514 | 0.014 |
| Follow up examination after treatment = yes (%) | 276 (1.5) | 182 (1.1) | 94 (3.6) | <0.001 | 0.165 |
| Chemotherapy session for neoplasm = yes (%) | 9401 (50.3) | 7163 (44.5) | 2238 (86.5) | <0.001 | 0.987 |
| Other chemotherapy = yes (%) | 7208 (38.6) | 6989 (43.4) | 219 (8.5) | <0.001 | 0.869 |
| Personal history of chemotherapy = yes (%) | 5703 (30.5) | 5359 (33.3) | 344 (13.3) | <0.001 | 0.486 |
| Convalescence following chemotherapy = yes (%) | 3 (0.0) | 3 (0.0) | 0 (0.0) | 1 | 0.019 |
| Cancer2 (%) |  |  |  | <0.001 | 0.546 |
| Bladder | 439 (2.3) | 409 (2.5) | 30 (1.2) |  |  |
| Brain | 203 (1.1) | 191 (1.2) | 12 (0.5) |  |  |
| Breast | 6638 (35.5) | 5703 (35.4) | 935 (36.2) |  |  |
| Colorectal | 2812 (15.0) | 2378 (14.8) | 434 (16.8) |  |  |
| Endometrium | 384 (2.1) | 287 (1.8) | 97 (3.8) |  |  |
| Esophagus | 500 (2.7) | 471 (2.9) | 29 (1.1) |  |  |
| Hodgkin | 225 (1.2) | 179 (1.1) | 46 (1.8) |  |  |
| Kidney | 159 (0.9) | 152 (0.9) | 7 (0.3) |  |  |
| Leukemia | 657 (3.5) | 591 (3.7) | 66 (2.6) |  |  |
| Liver | 86 (0.5) | 83 (0.5) | 3 (0.1) |  |  |
| Lung | 1105 (5.9) | 954 (5.9) | 151 (5.8) |  |  |
| Malignant melanoma | 383 (2.0) | 337 (2.1) | 46 (1.8) |  |  |
| Multiple myeloma | 537 (2.9) | 495 (3.1) | 42 (1.6) |  |  |
| Non-Hodgkin | 1484 (7.9) | 1234 (7.7) | 250 (9.7) |  |  |
| Oral | 568 (3.0) | 540 (3.4) | 28 (1.1) |  |  |
| Ovarian | 794 (4.2) | 486 (3.0) | 308 (11.9) |  |  |
| Pancreas | 331 (1.8) | 293 (1.8) | 38 (1.5) |  |  |
| Prostate | 993 (5.3) | 954 (5.9) | 39 (1.5) |  |  |
| Stomach | 309 (1.7) | 293 (1.8) | 16 (0.6) |  |  |
| Thyroid | 86 (0.5) | 77 (0.5) | 9 (0.3) |  |  |
| Delivery of a fraction of external beam radiotherapy NEC = yes (%) | 741 (4.0) | 458 (2.8) | 283 (10.9) | <0.001 | 0.324 |
| Megavoltage treatment for adaptive radiotherapy = yes (%) | 100 (0.5) | 86 (0.5) | 14 (0.5) | 1 | 0.001 |
| Oral delivery of radiotherapy for thyroid ablation = yes (%) | 23 (0.1) | 23 (0.1) | 0 (0.0) | 0.105 | 0.053 |
| Preparation for simple radiotherapy with imaging and dosimetry = yes (%) | 36 (0.2) | 11 (0.1) | 25 (1.0) | <0.001 | 0.125 |
| Megavoltage treatment for simple radiotherapy = yes (%) | 239 (1.3) | 45 (0.3) | 194 (7.5) | <0.001 | 0.38 |
| Preparation for complex conformal radiotherapy = yes (%) | 132 (0.7) | 98 (0.6) | 34 (1.3) | <0.001 | 0.072 |
| Technical support for preparation for radiotherapy = yes (%) | 117 (0.6) | 81 (0.5) | 36 (1.4) | <0.001 | 0.092 |
| Unspecified external beam radiotherapy = yes (%) | 124 (0.7) | 102 (0.6) | 22 (0.9) | 0.257 | 0.025 |
| Unspecified preparation for external beam radiotherapy = yes (%) | 46 (0.2) | 30 (0.2) | 16 (0.6) | <0.001 | 0.068 |
| Delivery of a fraction of interstitial radiotherapy = yes (%) | 48 (0.3) | 46 (0.3) | 2 (0.1) | 0.083 | 0.049 |
| Preparation for simple radiotherapy with imaging and simple calculation = yes (%) | 164 (0.9) | 19 (0.1) | 145 (5.6) | <0.001 | 0.334 |
| Unspecified support for preparation for radiotherapy = yes (%) | 15 (0.1) | 12 (0.1) | 3 (0.1) | 0.751 | 0.013 |
| Preparation for superficial radiotherapy with simple calculation= yes (%) | 6 (0.0) | 3 (0.0) | 3 (0.1) | 0.048 | 0.038 |
| Unspecified radiotherapy delivery = yes (%) | 100 (0.5) | 79 (0.5) | 21 (0.8) | 0.053 | 0.04 |
| Delivery of a fraction of intracavitary radiotherapy = yes (%) | 83 (0.4) | 60 (0.4) | 23 (0.9) | <0.001 | 0.065 |
| Other specified radiotherapy delivery = yes (%) | 50 (0.3) | 44 (0.3) | 6 (0.2) | 0.864 | 0.008 |
| Superficial or orthovoltage treatment for radiotherapy = yes (%) | 5 (0.0) | 3 (0.0) | 2 (0.1) | 0.295 | 0.027 |
| Megavoltage treatment for hypofractionated stereotactic radiotherapy = yes (%) | 8 (0.0) | 6 (0.0) | 2 (0.1) | 0.687 | 0.017 |
| Other specified support for preparation for radiotherapy = yes (%) | 3 (0.0) | 3 (0.0) | 0 (0.0) | 1 | 0.019 |
| Lipid-lowering medication = yes (%) | 1645 (8.8) | 1317 (8.2) | 328 (12.7) | <0.001 | 0.148 |
| Anti-hypertensive medication = yes (%) | 1488 (8.0) | 1025 (6.4) | 463 (17.9) | <0.001 | 0.359 |
| Anti-diabetic medication = yes (%) | 167 (0.9) | 120 (0.7) | 47 (1.8) | <0.001 | 0.095 |

Abbreviation: BMI, Body mass index; CRP, C-Reactive Protein; HDL, High Density Protein; HbA1c, haemoglobin A1c; MET, metabolic equivalent of task.

^1^ Continuous variables presented as mean (standard deviation); categorical variables as n (%).

**Supplementary Table S6. Bootstrap sensitivity analysis of cluster pairs based on Jaccard Index**

| Cluster A | Cluster B | Jaccard similarity index | Jaccard distance index |
| --- | --- | --- | --- |
| Cluster 1 | Cluster 2 | <0.001 | 1.000 |
| Cluster 1 | Cluster 3 | <0.001 | 1.000 |
| Cluster 1 | Cluster 4 | <0.001 | 1.000 |
| Cluster 1 | Cluster 5 | <0.001 | 1.000 |
| Cluster 1 | Cluster 6 | <0.001 | 1.000 |
| Cluster 1 | Cluster 7 | <0.001 | 1.000 |
| Cluster 1 | Cluster 8 | <0.001 | 1.000 |
| Cluster 1 | Cluster 9 | <0.001 | 1.000 |
| Cluster 2 | Cluster 1 | <0.001 | 1.000 |
| Cluster 2 | Cluster 3 | <0.001 | 1.000 |
| Cluster 2 | Cluster 4 | <0.001 | 1.000 |
| Cluster 2 | Cluster 5 | <0.001 | 1.000 |
| Cluster 2 | Cluster 6 | <0.001 | 1.000 |
| Cluster 2 | Cluster 7 | <0.001 | 1.000 |
| Cluster 2 | Cluster 8 | <0.001 | 1.000 |
| Cluster 2 | Cluster 9 | <0.001 | 1.000 |
| Cluster 3 | Cluster 1 | <0.001 | 1.000 |
| Cluster 3 | Cluster 2 | <0.001 | 1.000 |
| Cluster 3 | Cluster 4 | <0.001 | 1.000 |
| Cluster 3 | Cluster 5 | <0.001 | 1.000 |
| Cluster 3 | Cluster 6 | <0.001 | 1.000 |
| Cluster 3 | Cluster 7 | <0.001 | 1.000 |
| Cluster 3 | Cluster 8 | <0.001 | 1.000 |
| Cluster 3 | Cluster 9 | <0.001 | 1.000 |
| Cluster 4 | Cluster 1 | <0.001 | 1.000 |
| Cluster 4 | Cluster 2 | <0.001 | 1.000 |
| Cluster 4 | Cluster 3 | <0.001 | 1.000 |
| Cluster 4 | Cluster 5 | <0.001 | 1.000 |
| Cluster 4 | Cluster 6 | <0.001 | 1.000 |
| Cluster 4 | Cluster 7 | <0.001 | 1.000 |
| Cluster 4 | Cluster 8 | <0.001 | 1.000 |
| Cluster 4 | Cluster 9 | <0.001 | 1.000 |
| Cluster 5 | Cluster 1 | <0.001 | 1.000 |
| Cluster 5 | Cluster 2 | <0.001 | 1.000 |
| Cluster 5 | Cluster 3 | <0.001 | 1.000 |
| Cluster 5 | Cluster 4 | <0.001 | 1.000 |
| Cluster 5 | Cluster 6 | <0.001 | 1.000 |
| Cluster 5 | Cluster 7 | <0.001 | 1.000 |
| Cluster 5 | Cluster 8 | <0.001 | 1.000 |
| Cluster 5 | Cluster 9 | <0.001 | 1.000 |
| Cluster 6 | Cluster 1 | <0.001 | 1.000 |
| Cluster 6 | Cluster 2 | <0.001 | 1.000 |
| Cluster 6 | Cluster 3 | <0.001 | 1.000 |
| Cluster 6 | Cluster 4 | <0.001 | 1.000 |
| Cluster 6 | Cluster 5 | <0.001 | 1.000 |
| Cluster 6 | Cluster 7 | <0.001 | 1.000 |
| Cluster 6 | Cluster 8 | <0.001 | 1.000 |
| Cluster 6 | Cluster 9 | <0.001 | 1.000 |
| Cluster 7 | Cluster 1 | <0.001 | 1.000 |
| Cluster 7 | Cluster 2 | <0.001 | 1.000 |
| Cluster 7 | Cluster 3 | <0.001 | 1.000 |
| Cluster 7 | Cluster 4 | <0.001 | 1.000 |
| Cluster 7 | Cluster 5 | <0.001 | 1.000 |
| Cluster 7 | Cluster 6 | <0.001 | 1.000 |
| Cluster 7 | Cluster 8 | <0.001 | 1.000 |
| Cluster 7 | Cluster 9 | <0.001 | 1.000 |
| Cluster 8 | Cluster 1 | <0.001 | 1.000 |
| Cluster 8 | Cluster 2 | <0.001 | 1.000 |
| Cluster 8 | Cluster 3 | <0.001 | 1.000 |
| Cluster 8 | Cluster 4 | <0.001 | 1.000 |
| Cluster 8 | Cluster 5 | <0.001 | 1.000 |
| Cluster 8 | Cluster 6 | <0.001 | 1.000 |
| Cluster 8 | Cluster 7 | <0.001 | 1.000 |
| Cluster 8 | Cluster 9 | <0.001 | 1.000 |
| Cluster 9 | Cluster 1 | <0.001 | 1.000 |
| Cluster 9 | Cluster 2 | <0.001 | 1.000 |
| Cluster 9 | Cluster 3 | <0.001 | 1.000 |
| Cluster 9 | Cluster 4 | <0.001 | 1.000 |
| Cluster 9 | Cluster 5 | <0.001 | 1.000 |
| Cluster 9 | Cluster 6 | <0.001 | 1.000 |
| Cluster 9 | Cluster 7 | <0.001 | 1.000 |
| Cluster 9 | Cluster 8 | <0.001 | 1.000 |

**Supplementary Table S7. Algorithm sensitivity analysis using NMI and ARI**

| Algorithm | NMI | ARI |
| --- | --- | --- |
| HDBSCAN | 1 | 1 |
| Kmeans | 0.84 | 0.705 |
| Hierarchical | 0.844 | 0.717 |
| PAM | 0.84 | 0.71 |

Abbreviations: NMI, Normalized mutual information; ARI, Adjusted Rand index; HDBSCAN, Hierarchical Density-Based Spatial Clustering of Applications with Noise; PAM, Partitioning Around Medoids.

**Supplementary Table S8.** **Top 10 SHAP features for cluster identification**

| Cluster | Feature | Mean ABS SHAP | Cluster | Feature | Mean ABS SHAP |
| --- | --- | --- | --- | --- | --- |
| 1 | Other chemotherapy | 0.337 | 6 | Sex:Male | 1.797 |
| 1 | Sex:Male | 0.242 | 6 | Chemotherapy session for neoplasm | 1.519 |
| 1 | Chemotherapy session for neoplasm | 0.151 | 6 | Other chemotherapy | 0.164 |
| 1 | Personal history of chemotherapy | 0.129 | 6 | Cancer:Breast | 0.155 |
| 1 | Creatinine | 0.030 | 6 | Creatinine | 0.111 |
| 1 | HDL Cholesterol | 0.016 | 6 | HDL Cholesterol | 0.061 |
| 1 | Cancer:Prostate | 0.011 | 6 | Personal history of chemotherapy | 0.053 |
| 1 | Cancer:Breast | 0.010 | 6 | Age | 0.020 |
| 1 | Smoke:Never | 0.007 | 6 | Townsend index | 0.019 |
| 1 | Cholesterol | 0.005 | 6 | Cholesterol | 0.017 |
| 2 | Sex:Male | 2.202 | 7 | Chemotherapy session for neoplasm | 1.409 |
| 2 | Other chemotherapy | 1.871 | 7 | Smoke:Previous | 1.348 |
| 2 | Chemotherapy session for neoplasm | 0.880 | 7 | Other chemotherapy | 0.979 |
| 2 | Cancer:Breast | 0.255 | 7 | Personal history of chemotherapy | 0.972 |
| 2 | Personal history of chemotherapy | 0.242 | 7 | Sex:Male | 0.878 |
| 2 | Creatinine | 0.166 | 7 | Smoke:Never | 0.161 |
| 2 | HDL Cholesterol | 0.110 | 7 | Creatinine | 0.038 |
| 2 | Cancer:Prostate | 0.034 | 7 | HDL Cholesterol | 0.034 |
| 2 | Lipid-lowering medication | 0.033 | 7 | Cancer:Breast | 0.028 |
| 2 | HbA1c concentration 0 | 0.028 | 7 | Cholesterol | 0.022 |
| 3 | Personal history of chemotherapy | 1.319 | 8 | Personal history of chemotherapy | 1.048 |
| 3 | Chemotherapy session for neoplasm | 1.309 | 8 | Chemotherapy session for neoplasm | 0.786 |
| 3 | Sex:Male | 0.976 | 8 | Sex:Male | 0.451 |
| 3 | Smoke:Never | 0.867 | 8 | Smoke:Previous | 0.388 |
| 3 | Other chemotherapy | 0.759 | 8 | Other chemotherapyyes | 0.270 |
| 3 | Smoke:Previous | 0.668 | 8 | Smoke:Never | 0.266 |
| 3 | Cancer:Breast | 0.492 | 8 | Cancer:Breast | 0.044 |
| 3 | Lipid-lowering medication | 0.177 | 8 | Creatinine | 0.036 |
| 3 | Creatinine | 0.114 | 8 | HDL Cholesterol | 0.033 |
| 3 | Race:others | 0.091 | 8 | Glucose concentration | 0.021 |
| 4 | Personal history of chemotherapy | 0.968 | 9 | Sex:Male | 0.865 |
| 4 | Chemotherapy session for neoplasm | 0.853 | 9 | Personal history of chemotherapy | 0.847 |
| 4 | Sex:Male | 0.759 | 9 | Chemotherapy session for neoplasm | 0.824 |
| 4 | Other chemotherapyyes | 0.630 | 9 | Other chemotherapy | 0.387 |
| 4 | Smoke:Previous | 0.468 | 9 | Cancer:Breast | 0.145 |
| 4 | Smoke:Never | 0.305 | 9 | Creatinine | 0.075 |
| 4 | Cancer:Breast | 0.155 | 9 | HDL Cholesterol | 0.050 |
| 4 | Lipid-lowering medication | 0.109 | 9 | HbA1c concentration | 0.017 |
| 4 | Creatinine | 0.059 | 9 | Glucose concentration | 0.014 |
| 4 | Race:others | 0.054 | 9 | Cancer:Prostate | 0.011 |
| 5 | Personal history of chemotherapy | 0.868 |  |  |  |
| 5 | Chemotherapy session for neoplasm | 0.649 |  |  |  |
| 5 | Sex:Male | 0.453 |  |  |  |
| 5 | Delivery of a fraction of external beam radiotherapy NEC | 0.031 |  |  |  |
| 5 | Creatinine | 0.025 |  |  |  |
| 5 | Cancer:Breast | 0.019 |  |  |  |
| 5 | HDL Cholesterol | 0.019 |  |  |  |
| 5 | Glucose concentration | 0.011 |  |  |  |
| 5 | Smoke:Previous | 0.009 |  |  |  |
| 5 | Age | 0.009 |  |  |  |

Abbreviation: ABS, Absolute value; SHAP, SHapley Additive exPlanations; HbA1c,haemoglobin A1c; HDL, High-density lipoprotein

**Supplementary Table S9. Cox regression analysis of CVD risk across clusters**

| **Term** | **HR CI** | ***P* value** | **Outcome** | **Term** | **HR CI** | ***P* value** | **Outcome** |
| --- | --- | --- | --- | --- | --- | --- | --- |
| Cluster1 | 3.04 (2.57–3.58) | ＜0.001 | Essential (primary) hypertension | Cluster1 | 8.88 (1.18–66.67) | 0.034 | Aortic aneurysm and dissection |
| Cluster2 | 3.65 (3.07–4.34) | ＜0.001 | Essential (primary) hypertension | Cluster2 | 40.56 (5.52–298.24) | ＜0.001 | Aortic aneurysm and dissection |
| Cluster4 | 1.37 (1.11–1.68) | 0.003 | Essential (primary) hypertension | Cluster4 | 2.06 (0.19–22.70) | 0.556 | Aortic aneurysm and dissection |
| Cluster5 | 2.34 (1.96–2.80) | ＜0.001 | Essential (primary) hypertension | Cluster5 | 7.37 (0.94–57.63) | 0.057 | Aortic aneurysm and dissection |
| Cluster6 | 2.93 (2.48–3.47) | ＜0.001 | Essential (primary) hypertension | Cluster6 | 14.30 (1.95–104.77) | 0.009 | Aortic aneurysm and dissection |
| Cluster7 | 1.94 (1.48–2.54) | ＜0.001 | Essential (primary) hypertension | Cluster7 | 0.00 (0.00–Inf) | 0.996 | Aortic aneurysm and dissection |
| Cluster8 | 1.15 (0.89–1.50) | 0.284 | Essential (primary) hypertension | Cluster8 | 0.00 (0.00–Inf) | 0.994 | Aortic aneurysm and dissection |
| Cluster9 | 1.78 (1.41–2.26) | ＜0.001 | Essential (primary) hypertension | Cluster9 | 11.20 (1.00–125.92) | 0.050 | Aortic aneurysm and dissection |
| Cluster1 | 4.18 (2.37–7.38) | ＜0.001 | Angina pectoris | Cluster1 | 3.92 (2.69–5.73) | ＜0.001 | IHD |
| Cluster2 | 7.63 (4.29–13.59) | ＜0.001 | Angina pectoris | Cluster2 | 7.97 (5.44–11.66) | ＜0.001 | IHD |
| Cluster4 | 1.38 (0.67–2.81) | 0.379 | Angina pectoris | Cluster4 | 1.44 (0.90–2.29) | 0.128 | IHD |
| Cluster5 | 2.58 (1.40–4.77) | 0.002 | Angina pectoris | Cluster5 | 2.95 (1.98–4.39) | ＜0.001 | IHD |
| Cluster6 | 4.41 (2.50–7.80) | ＜0.001 | Angina pectoris | Cluster6 | 5.26 (3.62–7.64) | ＜0.001 | IHD |
| Cluster7 | 0.82 (0.18–3.65) | 0.795 | Angina pectoris | Cluster7 | 1.44 (0.63–3.28) | 0.391 | IHD |
| Cluster8 | 0.93 (0.30–2.87) | 0.900 | Angina pectoris | Cluster8 | 2.21 (1.24–3.95) | 0.007 | IHD |
| Cluster9 | 2.21 (0.94–5.21) | 0.070 | Angina pectoris | Cluster9 | 3.20 (1.88–5.45) | ＜0.001 | IHD |
| Cluster1 | 3.18 (1.65–6.15) | 0.001 | Acute myocardial infarction | Cluster1 | 0.84 (0.43–1.65) | 0.614 | Hemorrhagic stroke |
| Cluster2 | 7.46 (3.86–14.43) | ＜0.001 | Acute myocardial infarction | Cluster2 | 1.08 (0.51–2.28) | 0.846 | Hemorrhagic stroke |
| Cluster4 | 1.39 (0.62–3.13) | 0.424 | Acute myocardial infarction | Cluster4 | 0.82 (0.35–1.90) | 0.645 | Hemorrhagic stroke |
| Cluster5 | 2.81 (1.40–5.62) | 0.004 | Acute myocardial infarction | Cluster5 | 1.25 (0.62–2.53) | 0.532 | Hemorrhagic stroke |
| Cluster6 | 5.49 (2.88–10.45) | ＜0.001 | Acute myocardial infarction | Cluster6 | 1.17 (0.61–2.26) | 0.641 | Hemorrhagic stroke |
| Cluster7 | 0.60 (0.08–4.72) | 0.629 | Acute myocardial infarction | Cluster7 | 0.85 (0.19–3.83) | 0.830 | Hemorrhagic stroke |
| Cluster8 | 1.71 (0.58–5.04) | 0.331 | Acute myocardial infarction | Cluster8 | 0.96 (0.31–3.03) | 0.947 | Hemorrhagic stroke |
| Cluster9 | 2.16 (0.78–5.99) | 0.139 | Acute myocardial infarction | Cluster9 | 1.78 (0.69–4.60) | 0.236 | Hemorrhagic stroke |
| Cluster1 | 1.22 (0.84–1.78) | 0.299 | Pulmonary embolism | Cluster1 | 3.23 (1.73–6.03) | ＜0.001 | Ischemic stroke |
| Cluster2 | 1.17 (0.76–1.78) | 0.479 | Pulmonary embolism | Cluster2 | 5.13 (2.71–9.73) | ＜0.001 | Ischemic stroke |
| Cluster4 | 2.02 (1.34–3.04) | 0.001 | Pulmonary embolism | Cluster4 | 2.35 (1.16–4.76) | 0.017 | Ischemic stroke |
| Cluster5 | 2.83 (1.94–4.11) | ＜0.001 | Pulmonary embolism | Cluster5 | 3.94 (2.07–7.49) | ＜0.001 | Ischemic stroke |
| Cluster6 | 2.92 (2.04–4.18) | ＜0.001 | Pulmonary embolism | Cluster6 | 4.47 (2.41–8.30) | ＜0.001 | Ischemic stroke |
| Cluster7 | 3.94 (2.48–6.27) | ＜0.001 | Pulmonary embolism | Cluster7 | 3.89 (1.60–9.46) | 0.003 | Ischemic stroke |
| Cluster8 | 2.61 (1.67–4.09) | ＜0.001 | Pulmonary embolism | Cluster8 | 2.93 (1.28–6.68) | 0.011 | Ischemic stroke |
| Cluster9 | 2.56 (1.63–4.03) | ＜0.001 | Pulmonary embolism | Cluster9 | 5.40 (2.58–11.31) | ＜0.001 | Ischemic stroke |
| Cluster1 | 2.36 (1.68–3.32) | ＜0.001 | Atrial fibrillation and flutter |  |  |  |  |
| Cluster2 | 3.84 (2.70–5.45) | ＜0.001 | Atrial fibrillation and flutter |  |  |  |  |
| Cluster4 | 1.44 (0.96–2.17) | 0.081 | Atrial fibrillation and flutter |  |  |  |  |
| Cluster5 | 2.43 (1.70–3.47) | ＜0.001 | Atrial fibrillation and flutter |  |  |  |  |
| Cluster6 | 4.17 (3.00–5.81) | ＜0.001 | Atrial fibrillation and flutter |  |  |  |  |
| Cluster7 | 2.89 (1.65–5.08) | ＜0.001 | Atrial fibrillation and flutter |  |  |  |  |
| Cluster8 | 2.47 (1.50–4.06) | ＜0.001 | Atrial fibrillation and flutter |  |  |  |  |
| Cluster9 | 4.67 (3.04–7.17) | ＜0.001 | Atrial fibrillation and flutter |  |  |  |  |

Abbreviation: CVD, Cardiovascular; HR, Hazard ratio; CI, Confidence Interval; IHD, Ischemic Heart Disease.

**Supplementary Table S10.** **Harrell’s C-index for predicted models**

| Model1 | Model2 |
| --- | --- |
| 0.611 | 0.625 |

**Supplementary Table S11. Model performance evaluation for CVD risk prediction at 3,5 and 10 years**

| **Time** | **AUC M1** | **AUC M2** | **Delta AUC** | ***P* value** |
| --- | --- | --- | --- | --- |
| 3 | 0.628 | 0.649 | 0.021 | ＜0.001 |
| 5 | 0.634 | 0.661 | 0.027 | ＜0.001 |
| 10 | 0.648 | 0.692 | 0.044 | ＜0.001 |

Abbreviation: CVD, Cardiovascular diseases; AUC, Area Under the Curve.

**Supplementary Table S12.** **Time-dependent AUC for CVD risk prediction models**

| Times | Model1 | Model2 | Delta AUC |
| --- | --- | --- | --- |
| 3 | 0.628 | 0.649 | 0.021 |
| 3.5 | 0.629 | 0.65 | 0.022 |
| 4 | 0.632 | 0.656 | 0.024 |
| 4.5 | 0.631 | 0.657 | 0.025 |
| 5 | 0.634 | 0.661 | 0.027 |
| 5.5 | 0.637 | 0.666 | 0.028 |
| 6 | 0.639 | 0.668 | 0.029 |
| 6.5 | 0.642 | 0.672 | 0.03 |
| 7 | 0.645 | 0.676 | 0.031 |
| 7.5 | 0.648 | 0.68 | 0.032 |
| 8 | 0.647 | 0.68 | 0.033 |
| 8.5 | 0.648 | 0.683 | 0.035 |
| 9 | 0.65 | 0.687 | 0.037 |
| 9.5 | 0.648 | 0.688 | 0.04 |
| 10 | 0.648 | 0.692 | 0.044 |

Abbreviation: CVD, Cardiovascular diseases; AUC, Area Under the Curve.

**Supplementary Table S13. Baseline characteristics of identified clusters after excluding sex from the clustering input variables**

| **Variable** | **Cluster 1** | **Cluster 2** | **Cluster 3** | **Cluster 4** | **Cluster 5** | **Cluster 6** |
| --- | --- | --- | --- | --- | --- | --- |
| CRP aliquot | 1.11 | 1.13 | 1.11 | 1.09 | 1.11 | 1.1 |
| Cancer | Bladder: 0%,  Brain: 0%,  Breast: 100%, Colorectal: 0%,Endometrium: 0%,  Esophagus: 0%,Hodgkin: 0%,  Kidney: 0%,  Leukemia: 0%,Liver: 0%,  Lung: 0%, Malignant melanoma: 0%, Multiple myeloma: 0%, Non-Hodgkin: 0%, Oral: 0%, Ovarian: 0%, Pancreas: 0%, Prostate: 0%,  Stomach: 0%, Thyroid: 0% | Bladder: 0%,  Brain: 0%, Breast: 100%, Colorectal: 0%, Endometrium: 0%, Esophagus: 0%,Hodgkin: 0%, Kidney: 0%, Leukemia: 0%,Liver: 0%, Lung: 0%, Malignant melanoma: 0%, Multiple myeloma: 0%, Non-Hodgkin: 0%, Oral: 0%, Ovarian: 0%, Pancreas: 0%, Prostate: 0%, Stomach: 0%, Thyroid: 0% | Bladder: 2.5%,Brain: 1.3%, Breast: 22.7%, Colorectal: 16.9%,  Endometrium: 2.4%, Esophagus: 3.1%, Hodgkin: 1%, Kidney: 1.2%, Leukemia: 4.2%, Liver: 0.6%, Lung: 7.8%, Malignant melanoma: 3.3%, Multiple myeloma: 3.4%, Non-Hodgkin: 8.7%, Oral: 3.6%, Ovarian: 4.8%,Pancreas: 2.1%,Prostate: 7.7%,Stomach: 1.6%,Thyroid: 0.8% | Bladder: 2.7%,Brain: 1.1%, Breast: 28%, Colorectal: 19.1%, Endometrium: 2.5%, Esophagus:  3.2%,  Hodgkin: 2.1%,Kidney: 0.9%, Leukemia: 3.9%,  Liver: 0.5%,  Lung: 7.1%,  Malignant melanoma: 0.8%, Multiple myeloma: 2.2%,  Non-Hodgkin: 7.4%,  Oral: 2.9%,  Ovarian: 5.9%,Pancreas: 2.3%,Prostate: 4.5%,Stomach: 2.6%,Thyroid: 0.5% | Bladder: 2.1%,Brain: 0.9%, Breast: 38.3%, Colorectal:  15.2%,  Endometrium: 1.9%,  Esophagus: 2.4%,  Hodgkin: 1.4%,Kidney: 0.7%, Leukemia:  3.4%,  Liver: 0.2%,  Lung: 4.6%,  Malignant melanoma: 0.9%, Multiple myeloma: 3.6%,  Non-Hodgkin: 10.4%,  Oral: 4.6%,  Ovarian: 4.2%,Pancreas: 0.9%,Prostate: 2.6%,Stomach: 1.3%,Thyroid: 0.2% | Bladder: 2.8%,Brain: 1.7%, Breast: 31.2%, Colorectal:  16.2%,  Endometrium: 2.4%,  Esophagus:  3.1%,  Hodgkin: 0.7%,Kidney: 1%,  Leukemia:  3.4%,  Liver: 0.9%,  Lung: 5.7%,  Malignant melanoma: 2.4%, Multiple myeloma: 3.2%,  Non-Hodgkin: 6.7%,  Oral: 3.1%,  Ovarian: 3.6%,Pancreas: 2.4%Prostate: 7.3%,Stomach: 1.7%,Thyroid: 0.4% |
| Chemotherapy session for neoplasm | no: 0%,  yes: 100% | no: 100%,  yes: 0% | no: 99.9%,  yes: 0.1% | no: 0.1%,  yes: 99.9% | no: 0.5%,  yes: 99.5% | no: 100%,  yes: 0% |
| Cholesterol | 5.98 | 5.99 | 5.81 | 5.82 | 5.9 | 5.92 |
| Creatinine | 64.01 | 63.54 | 69.86 | 71.33 | 69.85 | 69.65 |
| Follow up examination after treatment | no: 99.8%,  yes: 0.2% | no: 100%,  yes: 0% | no: 99.9%,  yes: 0.1% | no: 98.2%,  yes: 1.8% | no: 98%,  yes: 2% | no: 98.5%,  yes: 1.5% |
| Glucose concentration | 5.02 | 4.92 | 4.99 | 5.1 | 5.01 | 4.94 |
| HDL Cholesterol | 1.66 | 1.64 | 1.49 | 1.47 | 1.52 | 1.53 |
| HbA1c_concentration_0 | 35.04 | 34.37 | 35.4 | 36.13 | 35.39 | 34.81 |
| Other chemotherapy | no: 100%,  yes: 0% | no: 0%,  yes: 100% | no: 0%,  yes: 100% | no: 98.6%,  yes: 1.4% | no: 87.2%,  yes: 12.8% | no: 99.9%,  yes: 0.1% |
| Personal_history_of_chemotherapy | no: 100%,  yes: 0% | no: 100%,  yes: 0% | no: 96.5%,  yes: 3.5% | no: 98.6%,  yes: 1.4% | no: 0.1%,  yes: 99.9% | no: 0%,  yes: 100% |
| Age | 55.47 | 54.86 | 56.83 | 58.53 | 57.62 | 56.61 |
| Alcohol | Current: 100%,Never: 0%,  Previous: 0%, unknown: 0% | Current: 99.9%,Never: 0%,  Previous: 0%, unknown: 0.1% | Current: 87.6%,Never: 6.2%,  Previous: 5.9%,unknown: 0.3% | Current: 89.2%,Never: 5.4%,  Previous: 5.1%,unknown: 0.3% | Current: 91.9%,Never: 3.9%,  Previous: 4%, unknown: 0.2% | Current: 93.3%,Never: 3.9%,  Previous: 2.7%,unknown: 0.1% |
| Hypertension | no: 99.7%,  yes: 0.3% | no: 100%,  yes: 0% | no: 99.4%,  yes: 0.6% | no: 96.7%,  yes: 3.3% | no: 98.3%,  yes: 1.7% | no: 100%,  yes: 0% |
| BMI | 26.18 | 26.57 | 27.3 | 27.16 | 26.82 | 26.29 |
| Antidiabetic medication | no: 99.5%,  yes: 0.5% | no: 100%,  yes: 0% | no: 99.1%,  yes: 0.9% | no: 98.2%,  yes: 1.8% | no: 99.5%,  yes: 0.5% | no: 100%,  yes: 0% |
| Antihypertensive medication | no: 100%,  yes: 0% | no: 100%,  yes: 0% | no: 91%,  yes: 9% | no: 84.5%,  yes: 15.5% | no: 94%,  yes: 6% | no: 99.1%,  yes: 0.9% |
| Lipid-lowering medication | no: 100%,  yes: 0% | no: 100%,  yes: 0% | no: 89.2%,  yes: 10.8% | no: 86%,  yes: 14% | no: 94.3%,  yes: 5.7% | no: 94.9%,  yes: 5.1% |
| Race | others: 0%,  white: 100% | others: 0%,  white: 100% | others: 6.1%, white: 93.9% | others: 4.4%, white: 95.6% | others: 4.4%, white: 95.6% | others: 4.1%, white: 95.9% |
| Delivery of a fraction of external beam radiotherapy NEC | no: 99.9%, yes: 0.1% | no: 99.3%,  yes: 0.7% | no: 99.3%,  yes: 0.7% | no: 96.2%,  yes: 3.8% | no: 93.3%,  yes: 6.7% | no: 95.3%,  yes: 4.7% |
| Delivery of a fraction of interstitial radiotherapy | no: 100%,  yes: 0% | no: 100%,  yes: 0% | no: 99.9%,  yes: 0.1% | no: 99.7%,  yes: 0.3% | no: 99.6%,  yes: 0.4% | no: 99.4%,  yes: 0.6% |
| Delivery of a fraction of intracavitary radiotherapy | no: 99.9%,  yes: 0.1% | no: 100%,  yes: 0% | no: 99.9%,  yes: 0.1% | no: 99.4%,  yes: 0.6% | no: 99.2%,  yes: 0.8% | no: 99.1%,  yes: 0.9% |
| Megavoltage treatment for simple radiotherapy | no: 100%,  yes: 0% | no: 100%,  yes: 0% | no: 99.9%,  yes: 0.1% | no: 99.9%,  yes: 0.1% | no: 98.7%,  yes: 1.3% | no: 99.8%,  yes: 0.2% |
| Oral delivery of radiotherapy for thyroid ablation | no: 99.9%,  yes: 0.1% | no: 100%,  yes: 0% | no: 99.9%,  yes: 0.1% | no: 99.8%,  yes: 0.2% | no: 99.8%,  yes: 0.2% | no: 100%,  yes: 0% |
| Other specified radiotherapy delivery | no: 99.9%,  yes: 0.1% | no: 100%,  yes: 0% | no: 99.9%,  yes: 0.1% | no: 99.5%,  yes: 0.5% | no: 99.7%,  yes: 0.3% | no: 99.9%,  yes: 0.1% |
| Preparation for complex conformal radiotherapy | no: 100%,  yes: 0% | no: 99.7%,  yes: 0.3% | no: 99.8%,  yes: 0.2% | no: 99.4%,  yes: 0.6% | no: 98.8%,  yes: 1.2% | no: 98.3%,  yes: 1.7% |
| Preparation for simple radiotherapy with imaging and simplecalculation | no: 100%,  yes: 0% | no: 99.9%,  yes: 0.1% | no: 99.9%,  yes: 0.1% | no: 99.9%,  yes: 0.1% | no: 99.7%,  yes: 0.3% | no: 99.8%,  yes: 0.2% |
| Preparation for simple radiotherapy with imaging and dosimetry | no: 100%,  yes: 0% | no: 100%,  yes: 0% | no: 100%,  yes: 0% | no: 99.9%,  yes: 0.1% | no: 99.3%,  yes: 0.7% | no: 100%,  yes: 0% |
| Technicical support for preparation for radiotherapy | no: 100%,  yes: 0% | no: 99.9%,  yes: 0.1% | no: 99.8%,  yes: 0.2% | no: 99.5%,  yes: 0.5% | no: 99.2%,  yes: 0.8% | no: 98.6%,  yes: 1.4% |
| Unspecified radiotherapy delivery | no: 99.6%,  yes: 0.4% | no: 100%,  yes: 0% | no: 100%,  yes: 0% | no: 98.4%,  yes: 1.6% | no: 99.7%,  yes: 0.3% | no: 99.9%,  yes: 0.1% |
| Unspecified external beam radiotherapy | no: 100%,  yes: 0% | no: 100%,  yes: 0% | no: 99.9%,  yes: 0.1% | no: 99%,  yes: 1% | no: 98%,  yes: 2% | no: 99.6%,  yes: 0.4% |
| Unspecified preparation for external beam radiotherapy | no: 100%,  yes: 0% | no: 100%,  yes: 0% | no: 100%,  yes: 0% | no: 99.7%,  yes: 0.3% | no: 99.4%,  yes: 0.6% | no: 99.9%,  yes: 0.1% |
| Sleep duration | 7.24 | 7.07 | 6.98 | 7.16 | 7.15 | 7.13 |
| Smoke | Current: 0%,  Never: 99.6%, Previous: 0%, unknown: 0.4% | Current: 0%,  Never: 99.2%, Previous: 0%, unknown: 0.8% | Current: 15%, Never: 43.6%, Previous:40.8%, unknown: 0.6% | Current: 14.3%,Never: 38.8%, Previous:46.5%,unknown: 0.4% | Current: 11.6%,Never: 49.8%, Previous:38.2%,unknown: 0.4% | Current: 13.2%,Never: 52.8%, Previous:33.7%,unknown: 0.3% |
| Townsend index | -1.75 | -1.8 | -1.15 | -1.12 | -1.27 | -1.49 |

Abbreviation: UMAP:Uniform Manifold Approximation and Project;HDBSCAN, Hierarchical Density-Based Spatial Clustering of Applications with Noise ;CRP,C-Reactive Protein; HDL,High-density lipoprotein ;Hb A1c,haemoglobin A1c;BMI,Body mass index

**Supplementary Table S14. Hazard ratios for specific cardiovascular outcomes across clusters after excluding sex from the clustering input variables**

| **Term** | **HR_CI** | ***P*.value** | **Outcome** | **Term** | **HR_CI** | **.value** | **Outcome** |
| --- | --- | --- | --- | --- | --- | --- | --- |
| cluster 1 | 0.66 (0.55–0.81) | <0.001 | Essential (primary) hypertension | cluster 1 | 0.40 (0.25–0.65) | <0.001 | IHD |
| cluster 2 | 1.34 (1.12–1.60) | 0.001 | Essential (primary) hypertension | cluster 2 | 1.20 (0.81–1.79) | 0.357 | IHD |
| cluster 3 | 2.24 (1.99–2.52) | <0.001 | Essential (primary) hypertension | cluster 3 | 2.29 (1.74–3.02) | <0.001 | IHD |
| cluster 4 | 1.66 (1.47–1.88) | <0.001 | Essential (primary) hypertension | cluster 4 | 1.31 (0.99–1.75) | 0.061 | IHD |
| cluster 5 | 1.73 (1.51–1.97) | <0.001 | Essential (primary) hypertension | cluster 5 | 1.84 (1.37–2.47) | <0.001 | IHD |
| cluster 1 | 0.69 (0.33–1.44) | 0.324 | Angina pectoris | cluster 1 | 0.75 (0.32–1.75) | 0.510 | hemorrhagic stroke |
| cluster 2 | 1.84 (0.97–3.49) | 0.062 | Angina pectoris | cluster 2 | 0.24 (0.05–1.08) | 0.063 | hemorrhagic stroke |
| cluster 3 | 3.65 (2.26–5.92) | <0.001 | Angina pectoris | cluster 3 | 0.85 (0.45–1.60) | 0.617 | hemorrhagic stroke |
| cluster 4 | 2.20 (1.35–3.60) | 0.002 | Angina pectoris | cluster 4 | 0.76 (0.40–1.44) | 0.399 | hemorrhagic stroke |
| cluster 5 | 2.34 (1.40–3.91) | 0.001 | Angina pectoris | cluster 5 | 1.16 (0.60–2.25) | 0.652 | hemorrhagic stroke |
| cluster 1 | 0.46 (0.18–1.14) | 0.092 | Acute myocardial infarction | cluster 1 | 0.27 (0.14–0.53) | <0.001 | ischemic stroke |
| cluster 2 | 0.89 (0.37–2.14) | 0.802 | Acute myocardial infarction | cluster 2 | 0.37 (0.18–0.75) | 0.006 | ischemic stroke |
| cluster 3 | 2.94 (1.69–5.09) | <0.001 | Acute myocardial infarction | cluster 3 | 1.04 (0.74–1.45) | 0.827 | ischemic stroke |
| cluster 4 | 2.00 (1.14–3.50) | 0.015 | Acute myocardial infarction | cluster 4 | 0.83 (0.59–1.17) | 0.285 | ischemic stroke |
| cluster 5 | 2.35 (1.32–4.18) | 0.004 | Acute myocardial infarction | cluster 5 | 1.15 (0.80–1.65) | 0.440 | ischemic stroke |
| cluster 1 | 0.35 (0.23–0.51) | <0.001 | Pulmonary embolism | cluster 1 | 0.28 (0.03–3.15) | 0.303 | Aortic aneurysm and dissection |
| cluster 2 | 0.31 (0.19–0.50) | <0.001 | Pulmonary embolism | cluster 2 | 0.60 (0.05–6.65) | 0.676 | Aortic aneurysm and dissection |
| cluster 3 | 0.41 (0.33–0.52) | <0.001 | Pulmonary embolism | cluster 3 | 5.47 (1.32–22.65) | 0.019 | Aortic aneurysm and dissection |
| cluster 4 | 0.79 (0.65–0.97) | 0.025 | Pulmonary embolism | cluster 4 | 1.37 (0.31–5.96) | 0.677 | Aortic aneurysm and dissection |
| cluster 5 | 1.10 (0.89–1.37) | 0.375 | Pulmonary embolism | cluster 5 | 3.68 (0.86–15.85) | 0.080 | Aortic aneurysm and dissection |
| cluster 1 | 0.27 (0.18–0.40) | <0.001 | Atrial fibrillation and flutter |  |  |  |  |
| cluster 2 | 0.42 (0.28–0.63) | <0.001 | Atrial fibrillation and flutter |  |  |  |  |
| cluster 3 | 0.84 (0.68–1.05) | 0.128 | Atrial fibrillation and flutter |  |  |  |  |
| cluster 4 | 0.58 (0.46–0.73) | 0.000 | Atrial fibrillation and flutter |  |  |  |  |
| cluster 5 | 1.03 (0.82–1.30) | 0.799 | Atrial fibrillation and flutter |  |  |  |  |

Abbreviation: CVD, Cardiovascular; HR, Hazard ratio; CI, Confidence Interval; IHD, Ischemic Heart Disease.

**Supplementary Table S15. Baseline characteristics of identified clusters in the female subgroup**

| Variable | Cluster1 | Cluster2 | Cluster3 | Cluster4 | Cluster5 | Cluster6 | Cluster7 | Cluster8 | Cluster9 |
| --- | --- | --- | --- | --- | --- | --- | --- | --- | --- |
| Age | 56.12 | 56.83 | 55.68 | 57.66 | 57.34 | 57.78 | 55.02 | 53.93 | 57.25 |
| Race | others: 4.5%, white: 95.5% | others: 4.7%, white: 95.3% | others: 0%, white: 100% | others: 2.1%, white: 97.9% | others: 10.3%, white: 89.7% | others: 2.1%, white: 97.9% | others: 0%, white: 100% | others: 6.7%, white: 93.3% | others: 17.1%, white: 82.9% |
| Townsend index | -1.53 | -1.25 | -1.74 | -1.09 | -0.22 | -1.26 | -1.78 | 0.14 | -1.18 |
| BMI | 26.04 | 26.64 | 26.18 | 26.72 | 26.71 | 27.12 | 26.56 | 26.7 | 27.26 |
| Sleep duration | 7.15 | 7.11 | 7.24 | 7.19 | 7.03 | 7.06 | 7.07 | 6.85 | 6.94 |
| Smoke | Current: 11.5%, Never: 55.6%, Previous: 32.5%, Unknown: 0.4% | Current: 10.3%, Never: 53.6%, Previous: 35.7%, Unknown: 0.5% | Current: 0%, Never: 99.6%, Previous: 0%, Unknown: 0.4% | Current: 0%, Never: 0%, Previous: 99.8%, Unknown: 0.2% | Current: 56.7%, Never: 43.2%, Previous: 0%, Unknown: 0.1% | Current: 0%, Never: 0%, Previous: 99.9%, Unknown: 0.1% | Current: 0%, Never: 99.2%, Previous: 0%, Unknown: 0.8% | Current: 99.8%, Never: 0%, Previous: 0%, Unknown: 0.2% | Current: 0.1%, Never: 99.3%, Previous: 0%, Unknown: 0.6% |
| Alcohol | Current: 92.6%, Never: 4.6%, Previous: 2.6%, Unknown: 0.2% | Current: 90.7%, Never: 4.9%, Previous: 4.1%, Unknown: 0.2% | Current: 100%, Never: 0%, Previous: 0%, Unknown: 0% | Current: 93.8%, Never: 1.4%, Previous: 4.8%, Unknown: 0% | Current: 76.6%, Never: 12.5%, Previous: 10.9%, Unknown: 0% | Current: 90.6%, Never: 3.3%, Previous: 6.1%, Unknown: 0.1% | Current: 99.9%, Never: 0%, Previous: 0%, Unknown: 0.1% | Current: 90%, Never: 2%, Previous: 7.8%, Unknown: 0.2% | Current: 61.9%, Never: 24.9%, Previous: 13.2%, Unknown: 0% |
| Hypertension | No: 100%, Yes: 0% | No: 98.6%, Yes: 1.4% | No: 99.7%, Yes: 0.3% | No: 99.6%, Yes: 0.4% | No: 98.5%, Yes: 1.5% | No: 99.6%, Yes: 0.4% | No: 100%, Yes: 0% | No: 99.8%, Yes: 0.2% | No: 99%, Yes: 1% |
| Glucose concentration | 4.92 | 5.02 | 5.02 | 5.05 | 5.05 | 5.01 | 4.93 | 4.86 | 4.99 |
| HbA1c concentration | 34.83 | 35.34 | 35.12 | 35.48 | 36.68 | 35.06 | 34.38 | 35.91 | 35.63 |
| Creatinine | 63.96 | 64.27 | 63.81 | 64.28 | 63.58 | 64.05 | 63.5 | 62.7 | 63.44 |
| CRP aliquot | 1.11 | 1.12 | 1.1 | 1.11 | 1.12 | 1.12 | 1.13 | 1.13 | 1.13 |
| Cholesterol | 6.02 | 6 | 6 | 6.08 | 5.95 | 5.99 | 5.98 | 6 | 5.83 |
| HDL Cholesterol | 1.64 | 1.62 | 1.65 | 1.65 | 1.51 | 1.65 | 1.65 | 1.5 | 1.57 |
| Follow up examination after treatment | No: 98.9%, Yes: 1.1% | No: 98.5%, Yes: 1.5% | No: 99.7%, Yes: 0.3% | No: 98.8%, Yes: 1.2% | No: 99%, Yes: 1% | No: 99.9%, Yes: 0.1% | No: 99.9%, Yes: 0.1% | No: 100%, Yes: 0% | No: 99.9%, Yes: 0.1% |
| Chemotherapy session for neoplasm | No: 100%, Yes: 0% | No: 0.1%, Yes: 99.9% | No: 0%, Yes: 100% | No: 0%, Yes: 100% | No: 0%, Yes: 100% | No: 99.7%, Yes: 0.3% | No: 100%, Yes: 0% | No: 100%, Yes: 0% | No: 100%, Yes: 0% |
| Other chemotherapy | No: 99.8%, Yes: 0.2% | No: 88%, Yes: 12% | No: 100%, Yes: 0% | No: 99.9%, Yes: 0.1% | No: 99.7%, Yes: 0.3% | No: 0%, Yes: 100% | No: 0%, Yes: 100% | No: 0%, Yes: 100% | No: 0.1%, Yes: 99.9% |
| Personal history of chemotherapy | No: 0%, Yes: 100% | No: 0%, Yes: 100% | No: 100%, Yes: 0% | No: 100%, Yes: 0% | No: 99.9%, Yes: 0.1% | No: 96.6%, Yes: 3.4% | No: 100%, Yes: 0% | No: 97.1%, Yes: 2.9% | No: 93.3%, Yes: 6.7% |
| Cancer | Bladder: 0.9%, Brain: 1.4%, Breast: 47.8%, Colorectal: 12.9%, Endometrium: 3.7%, Esophagus: 1.4%, Hodgkin: 0.7%, Kidney: 0.7%, Leukemia: 2.7%, Liver: 0.7%, Lung: 5.7%, Malignant melanoma: 2.6%, Multiple myeloma: 2.9%, Non-Hodgkin: 5.2%, Oral: 1.8%, Ovarian: 5.5%, Pancreas: 2%, Prostate: 0%, Stomach: 1%, Thyroid: 0.5% | Bladder: 0.9%, Brain: 0.6%, Breast: 56.6%, Colorectal: 10.2%, Endometrium: 2.8%, Esophagus: 1.1%, Hodgkin: 0.8%, Kidney: 0.4%, Leukemia: 1.9%, Liver: 0.1%, Lung: 3.9%, Malignant melanoma: 1%, Multiple myeloma: 2.1%, Non-Hodgkin: 7.6%, Oral: 2.2%, Ovarian: 6.3%, Pancreas: 0.6%, Prostate: 0%, Stomach: 0.5%, Thyroid: 0.2% | Bladder: 0.9%, Brain: 1.1%, Breast: 91.8%, Colorectal: 0%, Endometrium: 0%, Esophagus: 0.9%, Hodgkin: 0%, Kidney: 0.9%, Leukemia: 0%, Liver: 0.3%, Lung: 0%, Malignant melanoma: 0%, Multiple myeloma: 1.7%, Non-Hodgkin: 0%, Oral: 0.7%, Ovarian: 0%, Pancreas: 0%, Prostate: 0%, Stomach: 0.9%, Thyroid: 0.7% | Bladder: 1.1%, Brain: 0.5%, Breast: 60.5%, Colorectal: 11.9%, Endometrium: 3.1%, Esophagus: 2%, Hodgkin: 1.2%, Kidney: 0.4%, Leukemia: 2.8%, Liver: 0.6%, Lung: 0.8%, Malignant melanoma: 0.9%, Multiple myeloma: 0.9%, Non-Hodgkin: 0.4%, Oral: 1.4%, Ovarian: 8.7%, Pancreas: 1.1%, Prostate: 0%, Stomach: 1.2%, Thyroid: 0.4% | Bladder: 0.4%, Brain: 0.3%, Breast: 43.3%, Colorectal: 15.8%, Endometrium: 11.2%, Esophagus: 2.1%, Hodgkin: 0.9%, Kidney: 0%, Leukemia: 0.6%, Liver: 0.3%, Lung: 8.4%, Malignant melanoma: 0.3%, Multiple myeloma: 0.6%, Non-Hodgkin: 4.1%, Oral: 1.3%, Ovarian: 8%, Pancreas: 1.6%, Prostate: 0%, Stomach: 0.7%, Thyroid: 0.1% | Bladder: 0.9%, Brain: 1.1%, Breast: 42.1%, Colorectal: 13.4%, Endometrium: 3.2%, Esophagus: 1.9%, Hodgkin: 0.3%, Kidney: 1.4%, Leukemia: 2.9%, Liver: 0.8%, Lung: 7.7%, Malignant melanoma: 2.9%, Multiple myeloma: 3.5%, Non-Hodgkin: 7.5%, Oral: 1.9%, Ovarian: 5%, Pancreas: 1.5%, Prostate: 0%, Stomach: 1.1%, Thyroid: 0.9% | Bladder: 1.1%, Brain: 1.3%, Breast: 94.2%, Colorectal: 0%, Endometrium: 0%, Esophagus: 0.1%, Hodgkin: 1.3%, Kidney: 0.2%, Leukemia: 0%, Liver: 0.5%, Lung: 0%, Malignant melanoma: 0%, Multiple myeloma: 0%, Non-Hodgkin: 0%, Oral: 0.1%, Ovarian: 0%, Pancreas: 0%, Prostate: 0%, Stomach: 1.1%, Thyroid: 0.1% | Bladder: 0.8%, Brain: 0.6%, Breast: 50.6%, Colorectal: 11.4%, Endometrium: 2.7%, Esophagus: 1.2%, Hodgkin: 1.4%, Kidney: 0.2%, Leukemia: 2%, Liver: 0.6%, Lung: 9.4%, Malignant melanoma: 1.6%, Multiple myeloma: 2.9%, Non-Hodgkin: 4.7%, Oral: 1.6%, Ovarian: 5.3%, Pancreas: 2%, Prostate: 0%, Stomach: 0.8%, Thyroid: 0.4% | Bladder: 0.7%, Brain: 0.9%, Breast: 38%, Colorectal: 10.2%, Endometrium: 1.7%, Esophagus: 1.6%, Hodgkin: 0.8%, Kidney: 0.5%, Leukemia: 5.1%, Liver: 0.5%, Lung: 15.2%, Malignant melanoma: 2.9%, Multiple myeloma: 1.4%, Non-Hodgkin: 3.8%, Oral: 0.8%, Ovarian: 13.3%, Pancreas: 1.4%, Prostate: 0.1%, Stomach: 0.8%, Thyroid: 0.2% |
| Delivery of a fraction of external beam radiotherapy NEC | No: 95.8%, Yes: 4.2% | No: 94%, Yes: 6% | No: 100%, Yes: 0% | No: 99.6%, Yes: 0.4% | No: 96.6%, Yes: 3.4% | No: 99.4%, Yes: 0.6% | No: 99.1%, Yes: 0.9% | No: 99%, Yes: 1% | No: 99.7%, Yes: 0.3% |
| Oral delivery of radiotherapy for thyroid ablation | No: 99.9%, Yes: 0.1% | No: 99.7%, Yes: 0.3% | No: 99.7%, Yes: 0.3% | No: 99.9%, Yes: 0.1% | No: 99.9%, Yes: 0.1% | No: 99.9%, Yes: 0.1% | No: 100%, Yes: 0% | No: 99.8%, Yes: 0.2% | No: 99.8%, Yes: 0.2% |
| Preparation for simple radiotherapy with imaging and dosimetry | No: 100%, Yes: 0% | No: 99.9%, Yes: 0.1% | No: 100%, Yes: 0% | No: 100%, Yes: 0% | No: 100%, Yes: 0% | No: 100%, Yes: 0% | No: 100%, Yes: 0% | No: 100%, Yes: 0% | No: 99.9%, Yes: 0.1% |
| Megavoltage treatment for simple radiotherapy | No: 99.8%, Yes: 0.2% | No: 99.4%, Yes: 0.6% | No: 100%, Yes: 0% | No: 99.9%, Yes: 0.1% | No: 99.3%, Yes: 0.7% | No: 99.9%, Yes: 0.1% | No: 100%, Yes: 0% | No: 100%, Yes: 0% | No: 99.8%, Yes: 0.2% |
| Preparation for complex conformal radiotherapy | No: 98.2%, Yes: 1.8% | No: 98.6%, Yes: 1.4% | No: 100%, Yes: 0% | No: 99.9%, Yes: 0.1% | No: 99.3%, Yes: 0.7% | No: 99.8%, Yes: 0.2% | No: 99.5%, Yes: 0.5% | No: 99.8%, Yes: 0.2% | No: 99.9%, Yes: 0.1% |
| Technical support for preparation for radiotherapy | No: 98.8%, Yes: 1.2% | No: 98.9%, Yes: 1.1% | No: 100%, Yes: 0% | No: 99.9%, Yes: 0.1% | No: 99.4%, Yes: 0.6% | No: 99.8%, Yes: 0.2% | No: 99.7%, Yes: 0.3% | No: 100%, Yes: 0% | No: 99.9%, Yes: 0.1% |
| Unspecified external beam radiotherapy | No: 99.8%, Yes: 0.2% | No: 98.3%, Yes: 1.7% | No: 100%, Yes: 0% | No: 99.5%, Yes: 0.5% | No: 99.4%, Yes: 0.6% | No: 99.9%, Yes: 0.1% | No: 100%, Yes: 0% | No: 99.6%, Yes: 0.4% | No: 100%, Yes: 0% |
| Unspecified preparation for external beam radiotherapy | No: 99.9%, Yes: 0.1% | No: 99.6%, Yes: 0.4% | No: 100%, Yes: 0% | No: 99.7%, Yes: 0.3% | No: 99.9%, Yes: 0.1% | No: 99.9%, Yes: 0.1% | No: 100%, Yes: 0% | No: 100%, Yes: 0% | No: 100%, Yes: 0% |
| Delivery of a fraction of interstitial radiotherapy | No: 99.7%, Yes: 0.3% | No: 99.6%, Yes: 0.4% | No: 100%, Yes: 0% | No: 99.9%, Yes: 0.1% | No: 100%, Yes: 0% | No: 100%, Yes: 0% | No: 100%, Yes: 0% | No: 100%, Yes: 0% | No: 100%, Yes: 0% |
| Preparation for simple radiotherapy with imaging and simple calculation | No: 99.8%, Yes: 0.2% | No: 99.6%, Yes: 0.4% | No: 100%, Yes: 0% | No: 100%, Yes: 0% | No: 99.9%, Yes: 0.1% | No: 99.9%, Yes: 0.1% | No: 99.9%, Yes: 0.1% | No: 100%, Yes: 0% | No: 99.9%, Yes: 0.1% |
| Unspecified radiotherapy delivery | No: 99.8%, Yes: 0.2% | No: 99.8%, Yes: 0.2% | No: 99.3%, Yes: 0.7% | No: 99%, Yes: 1% | No: 98.1%, Yes: 1.9% | No: 100%, Yes: 0% | No: 100%, Yes: 0% | No: 100%, Yes: 0% | No: 100%, Yes: 0% |
| Delivery of a fraction of intracavitary radiotherapy | No: 98.9%, Yes: 1.1% | No: 98.8%, Yes: 1.2% | No: 99.9%, Yes: 0.1% | No: 99.5%, Yes: 0.5% | No: 96.9%, Yes: 3.1% | No: 99.9%, Yes: 0.1% | No: 100%, Yes: 0% | No: 99.8%, Yes: 0.2% | No: 100%, Yes: 0% |
| Other specified radiotherapy delivery | No: 99.9%, Yes: 0.1% | No: 99.8%, Yes: 0.2% | No: 99.6%, Yes: 0.4% | No: 99.1%, Yes: 0.9% | No: 99.9%, Yes: 0.1% | No: 99.9%, Yes: 0.1% | No: 100%, Yes: 0% | No: 100%, Yes: 0% | No: 100%, Yes: 0% |
| Lipid-lowering medication | No: 96%, Yes: 4% | No: 94.9%, Yes: 5.1% | No: 100%, Yes: 0% | No: 93.4%, Yes: 6.6% | No: 86.9%, Yes: 13.1% | No: 93.5%, Yes: 6.5% | No: 100%, Yes: 0% | No: 92.5%, Yes: 7.5% | No: 86.6%, Yes: 13.4% |
| Antihypertensive medication | No: 99.2%, Yes: 0.8% | No: 94.2%, Yes: 5.8% | No: 100%, Yes: 0% | No: 99%, Yes: 1% | No: 87.9%, Yes: 12.1% | No: 93.6%, Yes: 6.4% | No: 100%, Yes: 0% | No: 96.5%, Yes: 3.5% | No: 85.5%, Yes: 14.5% |
| Antidiabetic medication | No: 100%, Yes: 0% | No: 99.6%, Yes: 0.4% | No: 99.4%, Yes: 0.6% | No: 99.7%, Yes: 0.3% | No: 97.6%, Yes: 2.4% | No: 99.6%, Yes: 0.4% | No: 100%, Yes: 0% | No: 100%, Yes: 0% | No: 99.5%, Yes: 0.5% |

Abbreviation: UMAP:Uniform Manifold Approximation and Project;HDBSCAN, Hierarchical Density-Based Spatial Clustering of Applications with Noise ;CRP,C-Reactive Protein; HDL,High-density lipoprotein ;Hb A1c,haemoglobin A1c;BMI,Body mass index

**Supplementary Table S16 Hazard ratios for specific cardiovascular outcomes across clusters in the female subgroup**

| Cluster | HR (95%CI) | *P* value | Outcome | Cluster | HR (95%CI) | *P* value | Outcome |
| --- | --- | --- | --- | --- | --- | --- | --- |
| cluster 1 | 1.57 (1.27–1.94) | <0.001 | Essential (primary) hypertension | cluster 1 | 2.01 (1.20–3.36) | 0.008 | IHD |
| cluster 2 | 2.31 (1.93–2.76) | <0.001 | Essential (primary) hypertension | cluster 2 | 2.86 (1.92–4.28) | <0.001 | IHD |
| cluster 4 | 1.28 (1.04–1.58) | 0.018 | Essential (primary) hypertension | cluster 4 | 1.61 (1.02–2.54) | 0.040 | IHD |
| cluster 5 | 2.30 (1.86–2.84) | <0.001 | Essential (primary) hypertension | cluster 5 | 2.51 (1.57–4.01) | <0.001 | IHD |
| cluster 6 | 3.13 (2.60–3.76) | <0.001 | Essential (primary) hypertension | cluster 6 | 3.53 (2.32–5.37) | <0.001 | IHD |
| cluster 7 | 2.15 (1.75–2.65) | <0.001 | Essential (primary) hypertension | cluster 7 | 2.72 (1.70–4.34) | <0.001 | IHD |
| cluster 8 | 3.04 (2.43–3.80) | <0.001 | Essential (primary) hypertension | cluster 8 | 6.16 (3.93–9.67) | <0.001 | IHD |
| cluster 9 | 3.89 (3.21–4.72) | <0.001 | Essential (primary) hypertension | cluster 9 | 3.44 (2.19–5.41) | <0.001 | IHD |
| cluster 1 | 1.21 (0.51–2.86) | 0.671 | Angina pectoris | cluster 1 | 0.82 (0.30–2.25) | 0.701 | hemorrhagic stroke |
| cluster 2 | 2.38 (1.28–4.43) | 0.006 | Angina pectoris | cluster 2 | 1.22 (0.60–2.48) | 0.586 | hemorrhagic stroke |
| cluster 4 | 1.30 (0.63–2.68) | 0.475 | Angina pectoris | cluster 4 | 0.98 (0.44–2.19) | 0.968 | hemorrhagic stroke |
| cluster 5 | 2.96 (1.49–5.89) | 0.002 | Angina pectoris | cluster 5 | 0.71 (0.25–2.02) | 0.521 | hemorrhagic stroke |
| cluster 6 | 3.78 (2.02–7.06) | <0.001 | Angina pectoris | cluster 6 | 1.09 (0.48–2.45) | 0.840 | hemorrhagic stroke |
| cluster 7 | 2.66 (1.33–5.36) | 0.006 | Angina pectoris | cluster 7 | 0.26 (0.06–1.18) | 0.081 | hemorrhagic stroke |
| cluster 8 | 5.79 (2.94–11.39) | <0.001 | Angina pectoris | cluster 8 | 0.99 (0.32–3.11) | 0.990 | hemorrhagic stroke |
| cluster 9 | 3.75 (1.93–7.29) | <0.001 | Angina pectoris | cluster 9 | 0.88 (0.33–2.34) | 0.790 | hemorrhagic stroke |
| cluster 1 | 1.53 (0.59–3.97) | 0.380 | Acute myocardial infarction | cluster 1 | 3.25 (1.57–6.72) | 0.001 | ischemic stroke |
| cluster 2 | 2.73 (1.36–5.49) | 0.005 | Acute myocardial infarction | cluster 2 | 3.88 (2.04–7.38) | <0.001 | ischemic stroke |
| cluster 4 | 1.99 (0.93–4.26) | 0.075 | Acute myocardial infarction | cluster 4 | 1.81 (0.87–3.79) | 0.112 | ischemic stroke |
| cluster 5 | 1.74 (0.72–4.18) | 0.216 | Acute myocardial infarction | cluster 5 | 3.35 (1.61–6.95) | 0.001 | ischemic stroke |
| cluster 6 | 2.88 (1.36–6.08) | 0.006 | Acute myocardial infarction | cluster 6 | 3.21 (1.60–6.40) | 0.001 | ischemic stroke |
| cluster 7 | 1.27 (0.48–3.36) | 0.630 | Acute myocardial infarction | cluster 7 | 1.36 (0.56–3.30) | 0.497 | ischemic stroke |
| cluster 8 | 8.00 (3.79–16.90) | <0.001 | Acute myocardial infarction | cluster 8 | 5.55 (2.64–11.65) | <0.001 | ischemic stroke |
| cluster 9 | 2.27 (0.97–5.31) | 0.057 | Acute myocardial infarction | cluster 9 | 3.68 (1.78–7.59) | <0.001 | ischemic stroke |
| cluster 1 | 3.24 (2.18–4.83) | <0.001 | Pulmonary embolism | cluster 1 | 0.00 (0.00–Inf) | 0.997 | Aortic aneurysm and dissection |
| cluster 2 | 2.78 (1.91–4.05) | <0.001 | Pulmonary embolism | cluster 2 | 7.57 (0.97–59.17) | 0.054 | Aortic aneurysm and dissection |
| cluster 4 | 2.07 (1.37–3.11) | 0.001 | Pulmonary embolism | cluster 4 | 3.14 (0.33–30.16) | 0.322 | Aortic aneurysm and dissection |
| cluster 5 | 1.80 (1.12–2.87) | 0.014 | Pulmonary embolism | cluster 5 | 3.72 (0.34–41.06) | 0.284 | Aortic aneurysm and dissection |
| cluster 6 | 1.21 (0.77–1.91) | 0.405 | Pulmonary embolism | cluster 6 | 11.67 (1.43–95.43) | 0.022 | Aortic aneurysm and dissection |
| cluster 7 | 0.94 (0.55–1.60) | 0.811 | Pulmonary embolism | cluster 7 | 2.18 (0.13–35.49) | 0.583 | Aortic aneurysm and dissection |
| cluster 8 | 1.15 (0.63–2.12) | 0.647 | Pulmonary embolism | cluster 8 | 23.82 (2.81–202.18) | 0.004 | Aortic aneurysm and dissection |
| cluster 9 | 1.68 (1.05–2.69) | 0.029 | Pulmonary embolism | cluster 9 | 6.77 (0.69–66.24) | 0.100 | Aortic aneurysm and dissection |
| cluster 1 | 3.34 (2.18–5.11) | <0.001 | Atrial fibrillation and flutter |  |  |  |  |
| cluster 2 | 2.47 (1.72–3.53) | <0.001 | Atrial fibrillation and flutter |  |  |  |  |
| cluster 4 | 1.47 (0.98–2.21) | 0.063 | Atrial fibrillation and flutter |  |  |  |  |
| cluster 5 | 1.00 (0.59–1.68) | 0.990 | Atrial fibrillation and flutter |  |  |  |  |
| cluster 6 | 2.77 (1.89–4.07) | <0.001 | Atrial fibrillation and flutter |  |  |  |  |
| cluster 7 | 1.50 (0.94–2.41) | 0.092 | Atrial fibrillation and flutter |  |  |  |  |
| cluster 8 | 2.23 (1.35–3.69) | 0.002 | Atrial fibrillation and flutter |  |  |  |  |
| cluster 9 | 2.33 (1.52–3.58) | <0.001 | Atrial fibrillation and flutter |  |  |  |  |

Abbreviation: CVD, Cardiovascular; HR, Hazard ratio; CI, Confidence Interval; IHD, Ischemic Heart Disease.

**Supplementary Table S17. Baseline characteristics of identified clusters in the male subgroup**

| Variable | Cluster 1 | Cluster 2 | Cluster 3 |
| --- | --- | --- | --- |
| Age | 59.45 | 56.36 | 57.62 |
| Race | others: 3.3%, white: 96.7% | others: 5.3%, white: 94.7% | others: 3.3%, white: 96.7% |
| Townsend index | -1.28 | -1.14 | -1.45 |
| BMI | 27.34 | 27.69 | 26.73 |
| Sleep duration | 7.21 | 6.99 | 7.1 |
| Smoke | Current: 15.8%, Never: 40.1%, Previous: 43.6%, Unknown: 0.5% | Current: 16.2%, Never: 42.3%, Previous: 41%, Unknown: 0.5% | Current: 16.2%, Never: 47.1%, Previous: 36.6%, Unknown: 0.1% |
| Alcohol | Current: 93.3%, Never: 1.9%, Previous: 4.5%, Unknown: 0.3% | Current: 92.8%, Never: 2.9%, Previous: 4%, Unknown: 0.3% | Current: 94.5%, Never: 2.6%, Previous: 2.8%, Unknown: 0.1% |
| Hypertension | No: 96.9%, Yes: 3.1% | No: 99.5%, Yes: 0.5% | No: 100%, Yes: 0% |
| Glucose concentration | 5.08 | 5 | 4.98 |
| HbA1c concentration | 35.95 | 35.31 | 34.81 |
| Creatinine | 81.5 | 81 | 80.24 |
| CRP aliquot | 1.07 | 1.08 | 1.07 |
| Cholesterol | 5.63 | 5.6 | 5.73 |
| HDL Cholesterol | 1.3 | 1.28 | 1.33 |
| Follow up examination after treatment | No: 97.1%, Yes: 2.9% | No: 99.8%, Yes: 0.2% | No: 93.7%, Yes: 6.3% |
| Chemotherapy session for neoplasm | No: 0.5%, Yes: 99.5% | No: 98.1%, Yes: 1.9% | No: 100%, Yes: 0% |
| Other chemotherapy | No: 95%, Yes: 5% | No: 0%, Yes: 100% | No: 100%, Yes: 0% |
| Personal history of chemotherapy | No: 70.3%, Yes: 29.7% | No: 96.7%, Yes: 3.3% | No: 4.3%, Yes: 95.7% |
| Cancer | Bladder: 4.9%, Brain: 1.5%, Breast: 0.6%, Colorectal: 26.2%, Endometrium: 0%, Esophagus: 5.5%, Hodgkin: 2.5%, Kidney: 1.2%, Leukemia: 5.8%, Liver: 0.5%, Lung: 7.7%, Malignant melanoma: 1.8%, Multiple myeloma: 4.2%, Non-Hodgkin: 13.3%, Oral: 6.6%, Ovarian: 0%, Pancreas: 2.8%, Prostate: 10.5%, Stomach: 4.1%, Thyroid: 0.3% | Bladder: 5.3%, Brain: 1.7%, Breast: 0.5%, Colorectal: 19.8%, Endometrium: 0%, Esophagus: 4.9%, Hodgkin: 1.3%, Kidney: 1.1%, Leukemia: 7.2%, Liver: 0.4%, Lung: 7.1%, Malignant melanoma: 2.8%, Multiple myeloma: 4%, Non-Hodgkin: 10.6%, Oral: 6.1%, Ovarian: 0%, Pancreas: 2.7%, Prostate: 21.6%, Stomach: 2.6%, Thyroid: 0.2% | Bladder: 7.4%, Brain: 2.4%, Breast: 0.7%, Colorectal: 22.2%, Endometrium: 0%, Esophagus: 5.9%, Hodgkin: 0.7%, Kidney: 1.6%, Leukemia: 4.6%, Liver: 1.2%, Lung: 5.5%, Malignant melanoma: 2.2%, Multiple myeloma: 3.7%, Non-Hodgkin: 9.3%, Oral: 5.6%, Ovarian: 0%, Pancreas: 3.1%, Prostate: 20.8%, Stomach: 3%, Thyroid: 0.1% |
| Delivery of a fraction of external beam radiotherapy NEC | No: 93.2%, Yes: 6.8% | No: 98.9%, Yes: 1.1% | No: 94.5%, Yes: 5.5% |
| Oral delivery of radiotherapy for thyroid ablation | No: 99.9%, Yes: 0.1% | No: 99.8%, Yes: 0.2% | No: 100%, Yes: 0% |
| Preparation for simple radiotherapy with imaging and dosimetry | No: 99.8%, Yes: 0.2% | No: 100%, Yes: 0% | No: 99.7%, Yes: 0.3% |
| Megavoltage treatment for simple radiotherapy | No: 97.4%, Yes: 2.6% | No: 99.8%, Yes: 0.2% | No: 99.7%, Yes: 0.3% |
| Preparation for complex conformal radiotherapy | No: 99.3%, Yes: 0.7% | No: 99.8%, Yes: 0.2% | No: 98.5%, Yes: 1.5% |
| Technical support for preparation for radiotherapy | No: 99.2%, Yes: 0.8% | No: 99.8%, Yes: 0.2% | No: 98.3%, Yes: 1.7% |
| Unspecified external beam radiotherapy | No: 98.3%, Yes: 1.7% | No: 99.9%, Yes: 0.1% | No: 99.3%, Yes: 0.7% |
| Unspecified preparation for external beam radiotherapy | No: 99.5%, Yes: 0.5% | No: 100%, Yes: 0% | No: 99.8%, Yes: 0.2% |
| Delivery of a fraction of interstitial radiotherapy | No: 99.6%, Yes: 0.4% | No: 99.6%, Yes: 0.4% | No: 98.9%, Yes: 1.1% |
| Preparation for simple radiotherapy with imaging and simple calculation | No: 98.2%, Yes: 1.8% | No: 99.8%, Yes: 0.2% | No: 99.7%, Yes: 0.3% |
| Unspecified radiotherapy delivery | No: 98.6%, Yes: 1.4% | No: 100%, Yes: 0% | No: 100%, Yes: 0% |
| Delivery of a fraction of intracavitary radiotherapy | No: 100%, Yes: 0% | No: 99.9%, Yes: 0.1% | No: 99.7%, Yes: 0.3% |
| Other specified radiotherapy delivery | No: 99.3%, Yes: 0.7% | No: 100%, Yes: 0% | No: 99.9%, Yes: 0.1% |
| Lipid-lowering medication | No: 86.8%, Yes: 13.2% | No: 87.1%, Yes: 12.9% | No: 92.6%, Yes: 7.4% |
| Antihypertensive medication | No: 87.7%, Yes: 12.3% | No: 92.5%, Yes: 7.5% | No: 98.8%, Yes: 1.2% |
| Antidiabetic medication | No: 98.5%, Yes: 1.5% | No: 98.7%, Yes: 1.3% | No: 100%, Yes: 0% |

Abbreviation: UMAP:Uniform Manifold Approximation and Project;HDBSCAN, Hierarchical Density-Based Spatial Clustering of Applications with Noise ;CRP,C-Reactive Protein; HDL,High-density lipoprotein ;Hb A1c,haemoglobin A1c;BMI,Body mass index

**Supplementary Table S18. Hazard ratios for specific cardiovascular outcomes across clusters in the male subgroup**

| **Cluster** | **HR (95%CI)** | ***P* value** | **Outcome** |
| --- | --- | --- | --- |
| cluster1 | 1.73 (1.44–2.07) | <0.001 | Essential (primary) hypertension |
| cluster2 | 2.02 (1.68–2.43) | <0.001 | Essential (primary) hypertension |
| cluster1 | 2.40 (1.21–4.76) | 0.012 | Angina pectoris |
| cluster2 | 3.91 (1.98–7.74) | <0.001 | Angina pectoris |
| cluster1 | 2.44 (1.13–5.29) | 0.024 | Acute myocardial infarction |
| cluster2 | 3.22 (1.48–6.99) | 0.003 | Acute myocardial infarction |
| cluster1 | 1.26 (0.91–1.76) | 0.164 | Pulmonary embolism |
| cluster2 | 0.49 (0.33–0.72) | <0.001 | Pulmonary embolism |
| cluster1 | 1.03 (0.76–1.40) | 0.857 | Atrial fibrillation and flutter |
| cluster2 | 0.94 (0.68–1.30) | 0.713 | Atrial fibrillation and flutter |
| cluster1 | 1.76 (1.18–2.63) | 0.005 | IHD |
| cluster2 | 2.63 (1.76–3.92) | <0.001 | IHD |
| cluster1 | 0.69 (0.30–1.63) | 0.404 | hemorrhagic stroke |
| cluster2 | 0.74 (0.30–1.79) | 0.501 | hemorrhagic stroke |
| cluster1 | 0.92 (0.57–1.48) | 0.719 | ischemic stroke |
| cluster2 | 1.00 (0.61–1.64) | 0.995 | ischemic stroke |
| cluster1 | 1.00 (0.29–3.37) | 0.994 | Aortic aneurysm and dissection |
| cluster2 | 2.81 (0.85–9.22) | 0.089 | Aortic aneurysm and dissection |

Abbreviation: CVD, Cardiovascular; HR, Hazard ratio; CI, Confidence Interval; IHD, Ischemic Heart Disease.

**Supplementary Figure S1. Lasso coefficient paths and cross-validation curve**

(A) Coefficient Paths of the LASSO Cox Regression Model. (B) Cross-Validated Partial Likelihood Deviance for the LASSO Cox Regression Model.

Panel (A) shows the coefficient profiles of candidate variables plotted against the logarithm of the penalty parameter (logλ) in the LASSO Cox regression model. Each curve represents the trajectory of a regression coefficient as the penalty increases. Vertical dashed lines indicate the values of λ that minimize the partial likelihood deviance (λ min) and the largest value of λ within 1 standard error of the minimum (λ 1se). Panel (B) displays the cross-validated partial likelihood deviance as a function of logλ. Red points represent the mean deviance across folds, and error bars indicate ±1 standard error. The vertical dashed lines correspond to λ min and λ 1se, respectively.

**Supplementary Figure S2. Algorithmic clustering sensitivity analysis**

Abbreviations: ARI, Adjusted Rand index; NMI, Normalized mutual information; HDBSCAN, Hierarchical Density-Based Spatial Clustering of Applications with Noise; PAM, Partitioning Around Medoids.

**Supplementary Figure S3. LightGBM confusion matrix for cluster classification**

The confusion matrix displays the agreement between predicted and true class labels for the three-class classification model. Rows represent predicted classes, and columns represent true classes.

**Supplementary Figure S4.** Top predictive features for each patient cluster identified by SHAP analysis.

SHAP beeswarm plots displaying the most significant features driving the classification of Cluster 2-10 (A-I).

Each point represents an individual observation. The horizontal position indicates the SHAP value, reflecting the direction and magnitude of a given feature’s contribution to the model prediction for the corresponding cluster. Positive SHAP values indicate a higher predicted probability of belonging to the target cluster, whereas negative values indicate a lower predicted probability. Features are displayed on the y-axis in descending order of importance, defined by the mean absolute SHAP value. Point color denotes the original feature value, ranging from low (purple) to high (yellow).

Abbreviations: SHAP, SHapley Additive exPlanations; OVR, One-vs-Rest; NEC, not elsewhere classified; HbA1c, haemoglobin A1c; HDL, high-density lipoprotein; BMI, body mass index; TDI, Townsend deprivation index.

**Supplementary Figure S5. UMAP clustering and cardiovascular event distribution after excluding sex from the clustering input variables**

(A)The distribution of samples colored by identified clusters. (B) The distribution of samples colored by cardiovascular disease CVD event status.

**Supplementary Figure S6. Forest plot of hazard ratios for overall cardiovascular disease risk across clusters after excluding sex from the clustering input variables**

Hazard ratios (HRs) and 95% confidence intervals (CIs) for the study outcome according to cluster membership are shown, with Cluster 6 serving as the reference group. Estimates were derived from Cox proportional hazards regression models. Points indicate HRs, and horizontal bars represent 95% CIs. The vertical dashed line denotes an HR of 1. Values to the right of the plot report the HRs with corresponding 95% CIs and P values. The x-axis is presented on a logarithmic scale.

**Supplementary Figure S7. UMAP clustering and cardiovascular event distribution in the female subgroup**

(A) The distribution of samples colored by identified clusters. (B) The distribution of samples colored by cardiovascular disease CVD event status.

**Supplementary Figure S8. Forest plot of hazard ratios for overall cardiovascular disease risk across clusters in the female subgroup**

Hazard ratios (HRs) and 95% confidence intervals (CIs) for the study outcome according to cluster membership are shown, with Cluster 3 serving as the reference group. Estimates were derived from Cox proportional hazards regression models. Points indicate HRs, and horizontal bars represent 95% CIs. The vertical dashed line denotes an HR of 1. Values to the right of the plot report the HRs with corresponding 95% CIs and P values. The x-axis is presented on a logarithmic scale.

**Supplementary Figure S9. UMAP clustering and cardiovascular event distribution in the male subgroup**

(A) The distribution of samples colored by identified clusters. (B) The distribution of samples colored by cardiovascular disease CVD event status.

**Supplementary Figure S10. Forest plot of hazard ratios for overall cardiovascular disease risk across clusters in the male subgroup**

Hazard ratios (HRs) and 95% confidence intervals (CIs) for the study outcome according to cluster membership are shown, with Cluster 3 serving as the reference group. Estimates were derived from Cox proportional hazards regression models. Points indicate HRs, and horizontal bars represent 95% CIs. The vertical dashed line denotes an HR of 1. Values to the right of the plot report the HRs with corresponding 95% CIs and P values. The x-axis is presented on a logarithmic scale.
